# Isoenzyme-specific determination of glutathione peroxidase activity in cell lysates and GPX1/GPX4-HiBiT cellular target engagement

**DOI:** 10.1016/j.redox.2026.104445

**Published:** 2026-10-21

**Authors:** Madeleine S. Barrett, Jonathan H. Shrimp, Alina Löser, Dorian M. Cheff, Qing Cheng, Anna P. Kipp, Matthew D. Hall, Elias S.J. Arnér

**Affiliations:** a Division of Preclinical Innovation, National Center for Advancing Translational Sciences, National Institutes of Health, 9800 Medical Center Dr., Rockville, MD, 20850, USA; b Division of Biochemistry, Department of Medical Biochemistry and Biophysics, Karolinska Institutet, Stockholm, SE-171 77, Sweden; c Department of Nutritional Physiology, Institute of Nutritional Sciences, Friedrich Schiller University Jena, Dornburger Str. 24, Jena, 07743, Germany; d Assays, Profiling, and Cell Sciences, Discovery Sciences, BioPharmaceuticals R&D, AstraZeneca, Pepparedsleden 1, Mölndal, 431 83, Sweden; e Department of Selenoprotein Research and the National Tumor Biology Laboratory, National Institute of Oncology, 7 Ráth György utca, Budapest, 1122, Hungary

**Keywords:** Glutathione peroxidase, Auranofin, GPX lysate activity assay, Isoenzyme-specific, GPX1, GPX2, GPX4, HiBiT tagging, CETSA, Ferroptosis inducers, Target engagement

## Abstract

Glutathione peroxidases (GPX) are primarily selenoproteins that maintain cellular redox balance. GPX1, GPX2 and GPX4 are associated with cancer progression and treatment resistance, but the lack of isoenzyme-specific cellular assays has limited evaluation of GPX-targeting compounds. As GPX1 and GPX2 share high sequence homology, as well as substrate and inhibitor profiles, their individual activities are difficult to distinguish. Here, we present a glutathione reductase (GR)-coupled assay using auranofin to quantify combined GPX1/GPX2 activity and total GPX activity in crude cell lysates. The method was validated using multiple cell lines, with or without selenium supplementation, spiked human recombinant enzymes, and knockdown cells. Combined GPX1/GPX2 activity correlated with GPX1 protein levels and accounted for the majority of GPX activity in selenium-supplemented cells. GPX4's low catalytic turnover limits the feasibility of measuring its activity in cell lysates, so we established a high-throughput cellular thermal shift assay (CETSA) using HiBiT-tagging of GPX1 and GPX4 in HT1080 cells to quantify isoenzyme-specific target engagement. Using this new complementary assay platform, we found that ferroptosis inducers RSL3, PACMA-31 and ML-162 destabilized GPX4 in cells, RSL3 and ML-162 destabilized cellular GPX1, but none of these compounds had a strong effect on either total GPX, or GPX1/GPX2 activities in lysates. Additional reported GPX1/GPX2 or GPX4 inhibitors showed minimal GPX4 target engagement, whereas GPX1 was readily targeted in cells. The FDA-approved drugs, ceftibuten and omapatrilat, inhibited GPX1/GPX2 activity more potently than auranofin in cell lysates, suggesting that GPX1/GPX2 suppression may contribute to their cellular effects. Together, these complementary GPX assays presented here provide practical tools for evaluations of specific GPX isoenzyme cellular activities and development of selective GPX-targeted therapies.

## HIGHLIGHTS

• Complementary assays quantify GPX isoenzyme-specific lysate activity and target engagement.
• GPX1 accounts for most GPX activity in selenium-supplemented cancer cells.
• HiBiT-CETSA allows for efficient, isoenzyme-resolved target engagement in cells.
• GPX1 is more readily targeted than GPX4.
• GPX inhibitors differ in lysate inhibition and cellular target engagement.

## Graphical abstract

Made with Biorender.com.

**Figure:**
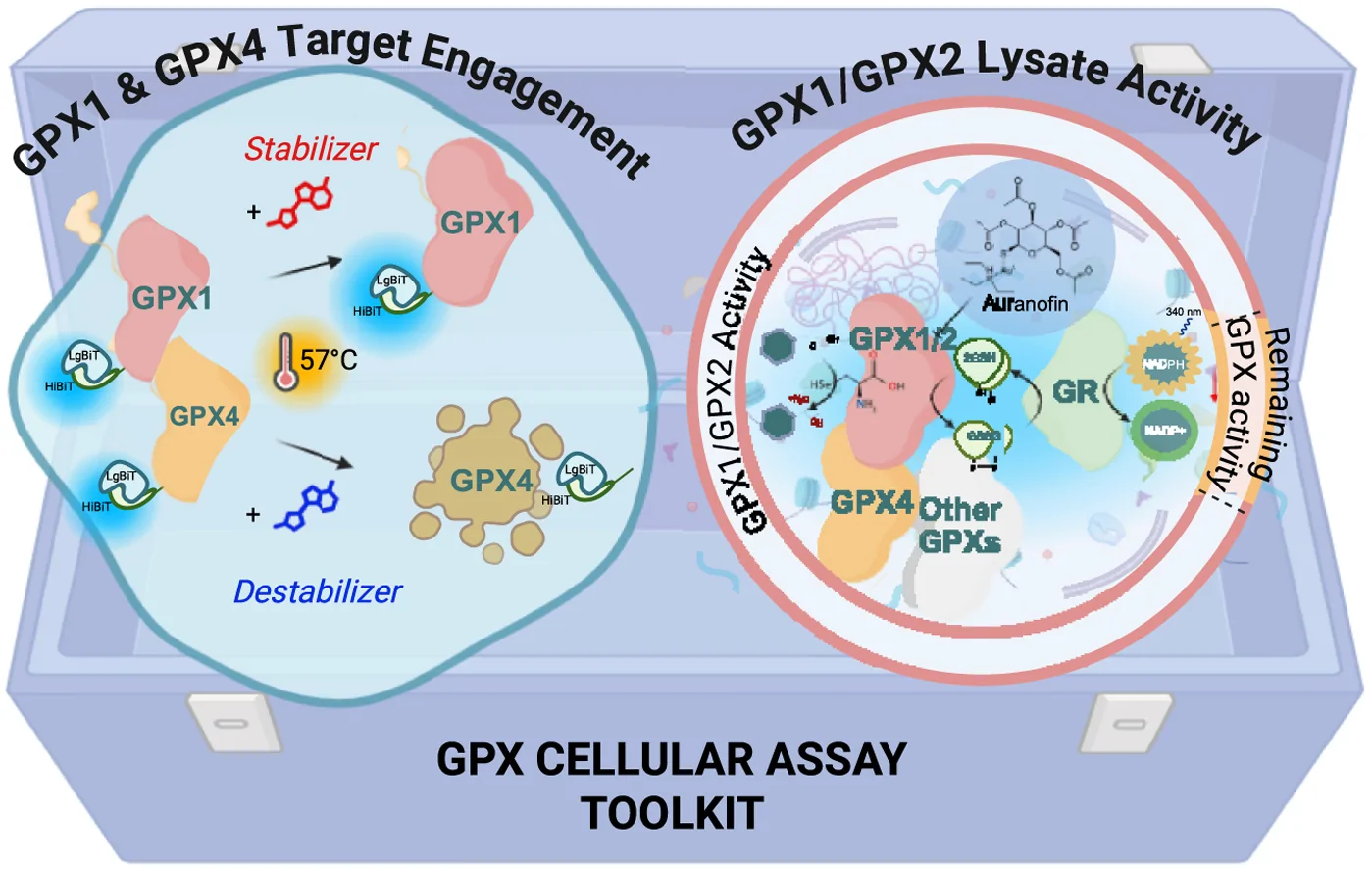

## 1 Introduction

Glutathione peroxidase (GPX) antioxidant enzymes are integral to a cell's ability to maintain redox homeostasis, acting as highly efficient peroxidases in concert with catalase and the thioredoxin system peroxiredoxins [1], [2]. Five of the eight human GPX enzymes are selenoproteins, containing a highly nucleophilic selenocysteine (Sec) residue in their active site [2], [3]. These selenium-dependent enzymes prevent downstream hydroxyl radical formation and oxidative damage by reducing hydroperoxides to their respective alcohol products. This reduction is coupled to the oxidation of two glutathione (GSH) molecules to form glutathione disulfide (GSSG) [4], [5], [6]. GSSG is subsequently recycled to two moieties of GSH by glutathione reductase (GR), and therefore GPX activity can be monitored in spectrophotometric assays through GR-coupled NADPH consumption. GPX enzymes often do not conform to classical Michaelis–Menten kinetics [2], [7], lacking a well-defined K_m_ or a saturable V_max_. Each GPX isoenzyme has a distinct substrate affinity, tissue distribution, subcellular localization and/or hierarchical selenium dependence, all of which may have important physiological and pathological implications to the roles of these enzymes in diverse cell types [2], [8], [9].

The first GPX isoenzyme discovered was GPX1 [2], [10], a homo-tetramer localized to the cytosol and potentially also the mitochondria [6], [11]. GPX1 is the most ubiquitously expressed isoenzyme in humans with peroxide reduction rates ∼20-fold and 10-fold higher than GPX2 and GPX4, respectively [9]. Its expression is highly dependent on cellular selenium levels; under selenium-deficient conditions, both its mRNA and protein levels decline rapidly due to nonsense-mediated decay of the mRNA, a short protein half-life and inefficient Sec insertion [12], [13], [14], [15], [16]. GPX1 may even function as a selenium-reserve when selenium is limited, allowing it to be preferentially recycled and incorporated into other selenoproteins such as thioredoxin reductase 1 (TrxR1) and GPX4 [17]. GPX4, a monomeric protein, ranks second most sensitive to selenium depletion among the GPXs, followed by GPX2, which shares >80% structural homology with GPX1 [18]. We recently found that GPX1 and GPX2 have closely overlapping specificities for both substrates [9] and inhibitors [19], which makes it difficult to distinguish between GPX1 and GPX2 isoenzyme-specific activities in cell or tissue lysates unless performing additional purification steps and/or immunological detection methods. The remaining GPX selenoprotein isoenzymes include GPX3, an extracellular isoenzyme secreted into the plasma [20], and GPX6, believed to be expressed mainly in the olfactory bulb [3]. The non-selenoprotein GPXs (GPX5, GPX7 and GPX8) may be misnomers, as they have been suggested not to be peroxidases using GSH as electron donor [21]. Therefore, in this study we have focused on developing methods for isoenzyme-specific analyses of GPX1, GPX2 and GPX4 in intact cells and crude cell lysates.

While all GPXs can reduce organic hydroperoxides, GPX4 is uniquely capable of reducing cholesteryl and phospholipid hydroperoxide substrates within cell membranes [9], [22], [23]. Disruption of GPX4 membrane association, including impaired binding to cardiolipin, compromises its biological function and causes the rare genetic disorder, Sedaghatian-type spondylometaphyseal dysplasia (SSMD), which is frequently neonatally lethal [24], [25]. Furthermore, GPX4 is the only GPX isoenzyme whose genetic deletion is embryonically lethal in mice [8], [26], highlighting its essential role detoxifying phospholipid peroxides in mammals.

Through its phospholipid peroxide-scavenging function, GPX4 acts as a pivotal regulator of ferroptosis, an iron-dependent, nonapoptotic form of cell death triggered by lipid peroxide accumulation [27], [28]. Treatment-resistant tumor cells, associated with poor clinical outcomes and known to generate high levels of lipid peroxides [29], were demonstrated to be uniquely reliant on GPX4 [30]. In addition, senescent tumor cells, known to promote tumor growth, also seem to be particularly dependent upon GPX4 [31]. As such, GPX4 is an attractive, though challenging, pharmacological target due to its shallow active site pocket and reduction of lipid substrates, typically requiring strong electrophiles or lipophilic compounds to bind to the Sec [31], [32], [33], [34]. GPX1 and GPX2 have also been implicated in disease progression and redox adaptation in several forms of cancer [35], [36], [37], [38], and GPX1 was identified as a promising collateral therapeutic target for multi-drug-resistant tumors [39], [40]. These isoenzyme-specific biochemical and pathophysiological differences highlight the need for methods capable of resolving GPX1/GPX2, and GPX4 activities and/or small molecule inhibitor engagements in cells.

Unlike previous studies that use hydroperoxide substrates, here we resolve isoenzyme-specific lysate activities using susceptibility to inhibition by the FDA-approved drug auranofin, primarily known as a potent thioredoxin reductase inhibitor [41], [42], [43], for its recent classification as a GPX1/GPX2 inhibitor that does not inhibit GPX4 or glutathione reductase (GR) [19]. Auranofin enables the selective determination of GPX1/GPX2 activities within the total GPX activity using stable reagents in crude cell lysates. Distinguishing between GPX1 and GPX2 isoenzyme activity is challenging for their aforementioned overlapping substrate [9] and inhibitor profiles [19], however the activities of other hydroperoxide-metabolizing enzymes such as catalase [44] are not detected in this assay for GPX activities with cumene hydroperoxide (CHP) as the substrate. CHP was selected because catalase is inefficient in reducing organic peroxides, whereas GPX1, GPX2, and GPX4 all efficiently catalyze CHP reduction [9], [45]. Peroxiredoxins (PRDX) [46] can also reduce cumene hydroperoxide [47], though sustained turnover of most mammalian peroxiredoxins depends primarily on the thioredoxin (TXN)/thioredoxin reductase (TrxR1) reducing system [48], not supplemented under these GR-coupled, GSH-dependent assay conditions. Therefore, their contribution to the activities detected in this assay is expected to be limited.

To complement this GPX1/GPX2-specific lysate assay, we developed an isoenzyme-resolved cellular thermal shift assay (CETSA) to specifically assess GPX1 (without measuring GPX2) or GPX4 target engagement in intact cells. Using CRISPR-Cas9 genome editing, we inserted an 11-amino acid peptide tag (termed HiBiT) [49] at the C-termini of either GPX1 or GPX4. HiBiT forms a functional luciferase upon complementation with LgBiT protein [50], enabling quantitative luminescent measurement [50] of either GPX1 or GPX4 protein levels and thermostability in live cells. Our HiBiT-based method is amenable for use in high-throughput screening projects and overcomes time-intensive limitations of traditional CETSA [51] that typically relies on lower-throughput antibody-based protein detection [49], [52]. Our CETSA approach also provides a new technique for measuring cellular GPX4-specific modulation as its enzymatic activity cannot be reliably detected in cell lysates [53]. Altogether, we present a complementary cellular assay platform for GPX1/GPX2-specific activity measurements using crude cell lysates and GPX1-and GPX4-HiBiT CETSA methodologies. Using this platform, we characterized, for the first time, a panel of GPX-directed modulators or small molecule inhibitory chemical probes in HT1080 cells.

## 2 Methods

### 2.1 Biochemical and cell biology methods

**Chemicals, reagents and compounds:** GPX assay buffer (TES) components: Tris(hydroxymethyl)aminomethane (Tris-HCl; Invitrogen, 15567-027), ethylenediaminetetraacetic acid (EDTA; Millipore Sigma, 324504), sodium chloride (NaCl; Thermo Scientific, J61807). Assay reagents: reduced l-glutathione (GSH, Thermo Fisher, A18014), cumene hydroperoxide (CHP; Sigma-Aldrich, 247502), β-nicotinamide adenine dinucleotide 2′-phosphate reduced tetrasodium salt hydrate (NADPH; Sigma-Aldrich, N7505), and bovine serum albumin (BSA; Sigma-Aldrich, A3311). Auranofin was purchased from Enzo (BML-EI206) and Cayman Chemical (15316). All annotated compounds tested were obtained from internal compound management sources at NIH/NCATS and were dissolved in dimethyl sulfoxide (DMSO; Fisher BioReagents, BP231). Lysate preparation components: PBS (Gibco, 10010023), Triton-x-100 (Thermo scientific, A16046), complete protease inhibitor cocktail (Roche, 11836170001).

**Cell cultures and lysate preparation:** Human cell lines: A549 (lung carcinoma), HEPG2 (hepatocellular carcinoma), HT1080 (fibrosarcoma) and MDA-MB-453 (metastatic carcinoma breast cancer), purchased from American Type Culture Collection (ATCC), were cultured in DMEM 4.5 g/L glucose medium (Gibco, 11965092) with 10% fetal bovine serum (FBS; Cytiva, SH30071) and 1% penicillin streptomycin (Gibco, 15140-122). Human HT-29 (colorectal adenocarcinoma) with stable knockdown of GPX1 and GPX2 (GPX1 KD, GPX2 KD) and control scramble (SCR) were generated as described previously [54], [55] and cultured in RPMI medium with 10% FBS, 1% PS and 1.1% GLUTAMAX. All selenium (selenite)-supplemented cell lines were cultured for at least 72 h in the presence of 100 nM sodium selenite, Na_2_SeO_3_ (SE; Sigma-Aldrich, 214485) to saturate GPX selenoprotein expression [56]. Cultures were maintained at 37°C in 5% CO_2_.

For lysate preparations, cells were trypsinized and collected from T175 flasks. After suspension in complete cell culture media, cells were resuspended in ice-cold PBS, centrifuging for 5 min at 1000 x *g* before each resuspension, with the cell pellet retained. The pellet was resuspended in 600 μL of lysis buffer (TES buffer, protease inhibitor cocktail and 0.1% v/v Triton X-100) and then subjected to three freeze-thaw cycles: 5 min on dry ice followed by 3 min on a heat block set to 50°C. Supernatants were retained after centrifuging at 4°C for 30 min at 15,000 x *g* and stored at −80°C until analysis. Protein concentration was determined using Promega Pierce bicinchoninic acid (BCA) assay kit.

**GPX1/GPX2 lysate activity assay:** First, crude cell lysate and a background control without lysate, were prepared with GR (50 nM), GSH (1 mM) and NADPH (500 μM) in TES buffer. All concentrations given are final [as in the well]. The lysate mixture containing GR/GSH/NADPH and cell lysate (30-50 μg/well) was briefly vortexed and incubated at room temperature (RT) for 15 min to pre-reduce and activate all GSH-dependent enzymes.

To determine GPX1/GPX2 activities as well as the total GPX activity in lysate, either 1 μL of auranofin (40 μM, in 0.4% v/v DMSO) or only DMSO (0.4% v/v) were added to wells of a clear, flat bottomed 96-well plate (Revvity), followed by 239 μL of the pre-incubated lysate mixture to the matched samples. Auranofin or DMSO were subsequently incubated with the lysate mixtures for another 15 min in the wells.

To measure specific GPX1/GPX2 activities and total GPX activity in lysates treated by other compounds of interest, 1 μl of compound or DMSO controls was added to wells followed by 188 μl of pre-incubated lysate mixture and incubated for 30 min at RT. Next, either 50 μl of auranofin (40 μM) or DMSO control in TES buffer was added to matching wells and incubated for 15 min (0.8% DMSO v/v).

Finally, 10 μL CHP (0.5 mM, in 70% w/v ethanol) was dispensed to wells (3.5% w/v ethanol final concentration), initiating the GPX activity reactions. Promptly, plates were shaken for 3 s, and NADPH absorbance (340 nm) was continuously read at 2-min intervals for 20-30 min on an Infinite M Nano plate reader (Tecan).

**Recombinant enzyme-spiked lysate assay:** Lysate samples were prepared as stated above (with GR, GSH, lysate and NADPH in TES buffer), with the addition of purified human recombinant selenocysteine GPX1, GPX4 or TrxR1 selenoproteins (produced as described previously [9], [19], [57], [58], [59]) using no-enzyme addition controls. Enzyme-spiked lysate samples were vortexed briefly and incubated for 15 min at RT before they were dispensed into wells with either auranofin (final concentration 40 μM), or DMSO (final concentration 0.8% v/v), and incubated for another 15 min. Upon the addition of CHP, NADPH absorbance was read at 2-min intervals, as described above.

To confirm recombinant TrxR1 activity, lysate samples were prepared with NADPH and 50 nM TrxR1 (plus a no enzyme control) in a 96-well plate pre-spotted with 1 μL compound (auranofin, TRi-1 or DMSO). Following a 30-min incubation, 100 μM sodium selenite was added to wells as a direct TrxR1 substrate [19], [60] and NADPH absorbance was read at 30-s intervals for 20 min.

**Activity assay analysis:** In the GPX1/GPX2 lysate activity assay, the rate of NADPH consumption was determined (linear rate in decrease of absorbance at 340 nm between 0 and 6 min, multiplied by −1), denoted as the total GPX activity in the DMSO-treated lysate samples. In the auranofin-treated samples, with GPX1/GPX2 activity inhibited, the NADPH consumption was similarly calculated and subsequently subtracted from the total GPX activity. As such, the difference between DMSO-treated lysate controls and auranofin-treated lysate samples was denoted as the GPX1/GPX2-derived GPX activity. The remaining activity that was uninhibited by auranofin was denoted as non-GPX1/GPX2 activity. The no lysate background control for non-specific NADPH oxidation was subtracted from all sample reads. To determine the absolute NADPH consumption per mg lysate, the rate of decrease in pathlength-corrected absorbance at 340 nm was converted to μmol NADPH consumed/min/mg protein using the Beer-Lambert equation (A = εlc) and the NADPH molar absorptivity at 340 nm, 6220 M^−1^cm^−1^ (ε). Percent GPX lysate activity was determined by normalizing NADPH Δ absorbance/min to DMSO-treated lysate and no lysate controls.

**Western Blots:** A total of 15 μl of lysate protein (10 μg) was denatured at 95°C with 4 μl LDS buffer (Novex Life Technologies) for 10 min. Denatured protein and buffer (4 μl of a protein ladder with molecular weights were dispensed in lane 1) were loaded into Bolt Plus 4-12% BisTris precast gels (Thermo Fisher Scientific) and separated by SDS-PAGE at 160 V for 40 min. Protein was dry transferred for 7 min at 20 V to a nitrocellulose membrane (iBlot 2) and blocked in 5% milk for 1 h. Membranes were then probed with primary antibodies for GPX1 (Abcam, AB22604), GPX2 (Abcam, AB137431), GPX4 (Abcam, AB125066 and Thermo Fisher, MA5-35089) or HiBiT (Promega, N7200) in TBST buffer (20 mM Tris, 150 mM NaCl and 0.1% Tween 20) diluted at 1:1000 overnight at 4°C. Following 3 × 5 min washes in TBST, membranes were probed for anti-rabbit or anti-mouse HRP-IgG (goat, Southern Biotech: 4030-05, 1030-05, 1:10,000) for 1 h at RT. Chemiluminescent signals were read using ECL detection reagent (Cytiva Amersham ImageQuant). Membranes were then washed 3x and the loading controls, β-Actin (Santa Cruz, 47778) and GAPDH (Thermo Fisher, MA5-15738-D488), were diluted to 1:10,000 and incubated with the membrane for 1 h at RT prior to chemiluminescent detection. Relative GPX1, GPX2 and GPX4 integrated protein density calculations were performed on Image J and normalized to the signal for β-Actin in each lane.

### 2.2 GPX1-and GPX4-HiBiT CRISPR tagging

RNP complex assembly and delivery: Ribonucleoprotein (RNP) complexes were assembled and electroporated into cells as previously described [49]. Briefly, 100 pmol of Alt-R S.p. Cas9 nuclease and 120 pmol of guide (gRNA) were mixed and incubated at ambient temperature for 10 min to form the RNP complex.

One million HT1080 cells were resuspended in 100 μl of 4D-Nucleofector Solution SF (Lonza Bioscience). The RNP complex, along with 200 pmol of single-stranded Alt-R HDR donor oligonucleotides containing the HiBiT sequence, was then electroporated into the cells using the 4D-Nucleofector System (Lonza Bioscience). Following electroporation, cells were incubated at ambient temperature for 10 min and then transferred to a six-well plate containing 2 mL of DMEM medium supplemented with 10% fetal bovine serum (Thermo Fisher Scientific). 48 h post-electroporation, cells were analyzed for HiBiT insertion using the Nano-Glo HiBiT Lytic Detection System containing LgBiT protein, for nanoLuciferase (NLuc) complex formation with HiBiT (1:1 vol addition per well), and luciferase substrate in a lytic buffer (Promega).

#### 2.2.1 Guide RNA (gRNA) and single stranded oligonucleotide (ssODN) sequences

##### 2.2.1.1 GPX1

Two gRNAs were evaluated for GPX1-tagging, with the sequence for C1 show in in Supplement and C2 gRNA sequence (20-mer) as follows: GGGGCGCCCTAGGCACAGCT

ssODN sequence (200-mer): CTACGCAGGTACAGCCGCCGCTTCCAGACCATTGACATCGAGCCTGACATCGAAGCCCTGCTGTCTCAAGGGCCCAGCTGTGCCGTGAGCGGCTGGCGGCTGTTCAAGAAGATTAGCTAGGGCGCCCCTCCTACCCCGGCTGCTTGGCAGTTGCAGTGCTGCTGTCTCGGGGGGGTTTTCATCTATGAGGGTGTTTCCTC.

##### 2.1.1.2 GPX4

Three gRNAs were evaluated for GPX4-tagging, with the sequences for C2 and C3 shown in Supplement and C1 gRNA sequence (20-mer) as follows: CGGGCGGGGCCACACACTTG

ssODN sequence (200-mer): GAGGCTCGGGGGCTTGGGAGGTAGCTGCCCTAACCCAGCTTTCCTCCCCGACAGGTGATAGAGAAGGACCTGCCCCACTATTTCGTGAGCGGCTGGCGGCTGTTCAAGAAGATTAGCTAGCTACACAAGTGTGTGGCCCCGCCCGAGCCCCTGCCCACGCCCTTGGAGCCTTCCACCGGCACTCATGACGGCCTGCCTGC.

Single-cell cloning: The GPX4-HiBiT HT1080 cells were suspended (500 cells/mL) in DMEM (10% FBS, 1% PS) dispensed ∼1 cell/well into a 384 plate (Corning, CellBIND, 3770) in 30 μl of medium. After two weeks of growth, wells were analyzed for confluency (Incucyte) and monoclonal populations were selected based on strength of NLuc from GPX4-HiBiT quantified using the Nano-Glo HiBiT Lytic Detection System (Promega) and Western blots for GPX4 protein as well as HiBiT tag levels.

**Melting temperature determination of GPX1 and GPX4-HiBiT**: 15 μL of monoclonal GPX1-HiBiT- or GPX4-HiBiT-expressing HT1080 cells (10,000 cells/well) in DMEM medium supplemented with 10% fetal bovine serum were transferred to a 384-well PCR plate (Roche) and heated on a thermocycler (Bio-Rad) for 3.5 min at various temperatures. Following heat treatment, GPX1-HiBiT or GPX4-HiBiT levels were quantified using the Nano-Glo HiBiT Lytic Detection System (Promega) and a melt temperature was chosen to be 55°C for GPX4-HiBiT and 60°C for GPX1-HiBiT CETSA.

**Cellular Thermal Shift Assay**: The cellular thermal shift assay (CETSA) was performed as previously described [61]. A 384-well PCR plate (Roche) was spotted with 60 nL of small molecules or DMSO as vehicle to each well. The HiBiT-inserted HT1080 cells were collected and suspended in DMEM (Cat#: 10566016) with 10% FBS. 10 μl of cells (10,000 cells/well) were dispensed using a multidrop combi (ThermoFisher) into a 384-well PCR plate (Roche) and incubated for 1 h at 37°C with 5% CO_2_. Each well was heated at the chosen temperature for each cell line on a thermocycler for 3.5 min. The cells were then lysed using the Nano-Glo HiBiT Lytic Detection System (Promega) to quantify the amount of GPX1-and GPX4-HiBiT.

### 2.3 CETSA analyses

For the CETSA experiments, the HiBiT-LgBiT luminescent signals for the compound-treated samples were normalized to the DMSO controls. Because assay responses are normalized to a zero-response baseline, with -100% representing maximal thermal destabilization and 100% representing maximal thermal stabilization, destabilization yields negative values. (*)AUC represents the integrated concentration-response across the tested concentration range in the CETSA analyses. More negative AUC values indicate a greater overall destabilizing response across the concentration range, reflecting both response magnitude and concentration dependence.

### 2.4 Statistical analyses

GraphPad Prism was used to graph results and perform statistical tests including two-way ANOVA with Tukey's multiple comparison post-hoc analysis where appropriate. All graphs and values are stated as mean ± standard deviation (SD). Simple linear regression and Pearson's correlation models were fitted where indicated; slopes were reported with 95% confidence intervals and correlation coefficients. Concentration-response curves were plotted on a logarithmic scale and fitted with a linear regression curve.

## 3 Results

### 3.1 Development of a GPX1/GPX2 isoenzyme-specific enzymatic assay compatible with crude cell lysates

The total GPX activity in a crude cell lysate is, by definition, the combined enzymatic GSH-dependent reduction of hydroperoxides to corresponding alcohols and GSSG. To detect this GPX activity in lysates, NADPH consumption is monitored as GR recycles GSSG to GSH. As we have previously reported, CHP is an ideal hydroperoxide substrate for this assay, conferring a lower background signal [19], [45]. Table 1 and Fig. 1A outline the stepwise protocol developed here to detect GPX1/GPX2 and total GPX activity in crude cell lysates.

**Table 1:** Protocol for the total GPX and specific GPX1/GPX2 activity assay using crude cell lysates.

Step	Reagents/Conditions		Final Assay Concentrations
**1**	**1^st^**15 min pre-incubation: GR, GSH and NADPH in TES buffer ± cell lysate. Prepared in tubes (volume = 250 μl x # of sample wells).		See step 3 for final concentrations.
**2**	Add **1 μL** GPX1/GPX2 inhibitor, **auranofin**, or DMSO control, to half of the wells in a 96-well plate		± auranofin = **40 μM**
**3**	Following the pre-incubation in Step 1, add **239 μl** of samples to multiple wells in a 96-well plate, with or without auranofin (see Step 2).	**Cell lysate***(with 0.1% Triton-100, protease inhibitor in lysis buffer)*	**30-50 μg total protein per well**
		**GR**	**50 nM**
		**NADPH**	**0.5 mM**
		**GSH**	**1 mM**
**4**	**2**^nd^15 min incubation w**/auranofin in wells**		
**5**	Add **10 μl CHP**^a^ to all wells to start the reaction		**0.5 mM**
**6**	**Read NADPH 340 nm absorbance** 15 × 2 min interval reads		

Notes.

All reagents in TES Buffer: 50 mM Tris-HCl, pH 7.5, 2 mM EDTA, 150 mM NaCl.

a CHP prepared in 70% ethanol; final assay concentrations of DMSO 0.8% v/v and of ethanol 3.5% w/v. **Final well volume = 250 μL**

**Fig. 1:**
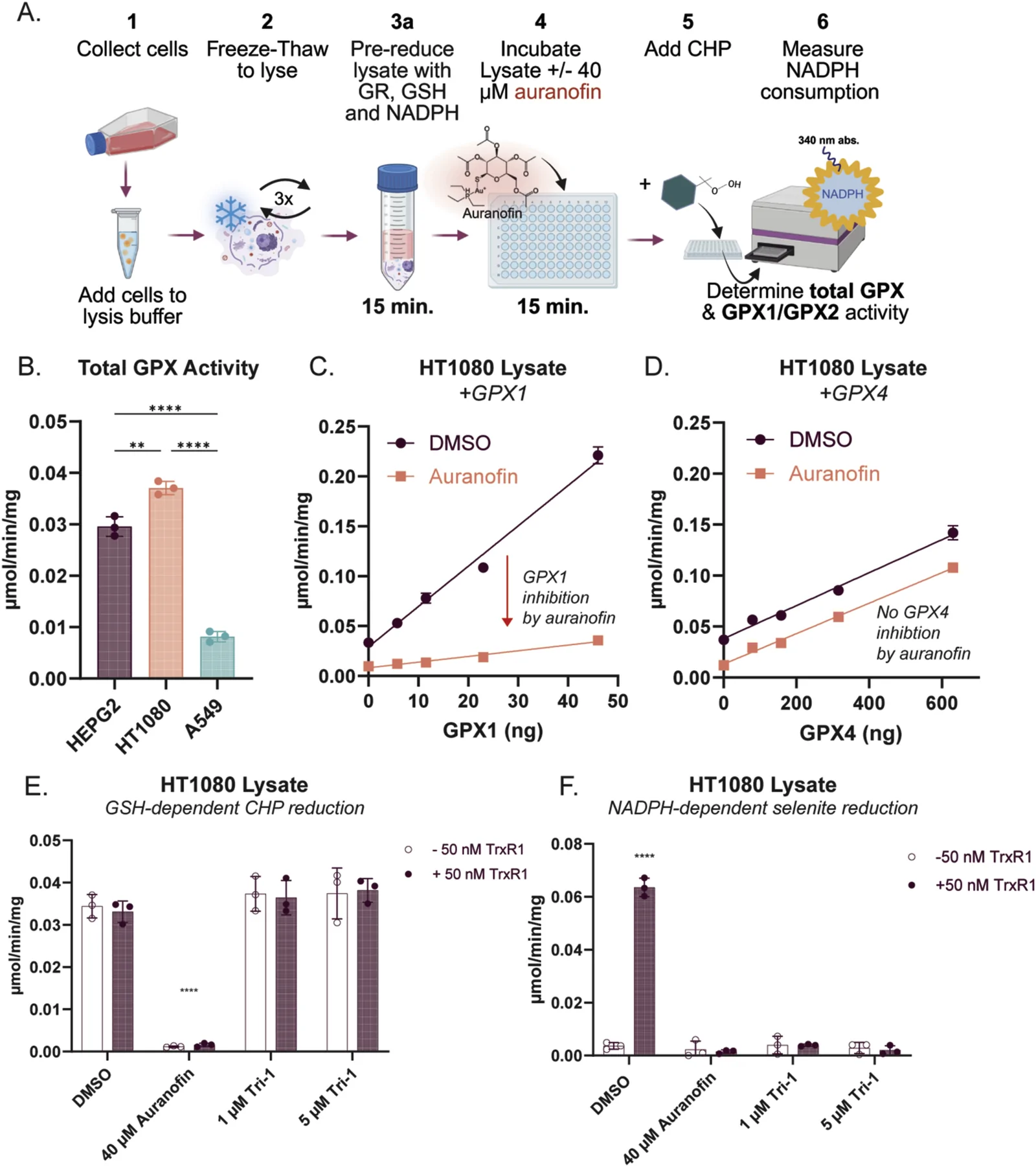
Cell line model selection and validation of GPX isoenzyme-specific activity detection. **(A)** Stepwise scheme showing the GPX1/GPX2 isoenzyme-specific lysate activity assay protocol developed here (also see Table 1). Created with Biorender.com. **(B)** Total GPX activity, reported as NADPH consumption (μmol NADPH consumed per minute per mg lysate), of lysates from three different cell lines (30 μg/well). ** = p < 0.01, **** = p < 0.001. Auranofin (40 μM) inhibits **(C)** total GPX activity and recombinant GPX1, but not **(D)** recombinant GPX4 activity spiked in 30 μg of lysate from HT1080 cells. For each condition, a linear regression curve was determined between NADPH consumption (both from HT1080 lysates and recombinant GPX1 or GPX4) and amount of spiked GPX protein (ng). NADPH consumption during **(E)** Glutathione (GSH)-dependent CHP reduction in HT1080 cell lysates via the GR-coupled assay and **(F)** NADPH-dependent sodium selenite (100 μM) reduction in the presence (solid bars and dots) or absence (white bars and open dots) of 50 nM recombinant human TrxR1, following treatment with auranofin, TRi-1, or DMSO vehicle control (0.8% v/v). For all figure panels, lysates came from cells that were selenium-supplemented, n = 3 technical replicates. ** = p < 0.01, **** = p < 0.001, compared to DMSO control.

First, we compared the total GPX activity in lysates from three cell lines (HEPG2 hepatocellular carcinoma, HT1080 fibrosarcoma, and A549 lung adenocarcinoma) commonly used in glutathione peroxidase and ferroptosis research [14], [27], [32], [53], [56], [62], [63]. Cells were cultured for at least 72 hours in 100 nM sodium selenite to saturate their endogenous selenoprotein synthesis [16], [56] prior to lysate preparation. Among these, HT1080 lysates had the highest total GPX activity (Fig. 1B). Because HT1080 cells are also widely used in ferroptosis research and GPX4-targeted small-molecule discovery [27], [32], [53], [62], we selected this model for the development of our GPX1/GPX2-specific lysate activity and subsequent CETSA studies.

Next, we assessed the selective inhibition of GPX1/GPX2 activity in lysates using auranofin, a direct inhibitor of both GPX1 and GPX2 (IC_50_ = 4.6 and 2.1 μM) that does not directly inhibit GPX4 or GR [19], [42]. We spiked increasing amounts of human recombinant GPX1 or GPX4 into selenium-supplemented lysates from HT1080 cells and measured total GPX and GPX1/GPX2 activities in the presence of auranofin (40 μM) or DMSO (Fig. 1C and D), following the assay protocol (Table 1 and Fig. 1A). Due to challenges in expressing recombinant, Sec-containing GPX1 and GPX4, the *E. coli* expression system used here results in a mixture of enzyme species: ∼20% Sec-containing GPX enzyme and ∼80% misincorporated or missing Sec (inactive) enzyme from errant Sec translation [58], [59]. As such, only the Sec-containing species are enzymatically active and detected using activity-based assays. The recombinant GPX1 and GPX4 enzyme amounts (ng) shown on the x-axes in Fig. 1C and D correspond to the active ∼20% Sec-containing fraction of the recombinant enzymes added to each lysate. Because GPX1 is substantially more active than GPX4 [9], [19], 15x less GPX1 protein was used to achieve comparable activity readouts. While the addition of increasing amounts of recombinant GPX enzymes to the lysates correspondingly increased the rates of NADPH consumption in the assay, auranofin only inhibited the GPX1-derived activity in the lysates at all recombinant protein levels and did not inhibit the activity of the recombinant GPX4 added to the lysate (Fig. 1C and D), consistent with prior reports [19]. These results demonstrate that by measuring GPX activity in cell lysates using the protocol given in Table 1 and Fig. 1A, the addition of auranofin blocks the activity derived from GPX1, but not that of GPX4.

Next, considering auranofin is also a potent inhibitor of TrxR1 (IC_50_ = 0.2 μM) [41], we sought to rule out interference from thioredoxin reductase 1 (TrxR1) in the GPX lysate assay. Though TrxR1, like GR, is an NADPH-dependent, redox-cycling enzyme, we did not suspect it to distort the GPX assay readout as it is an inefficient reductant of GSSG [64]. HT1080 lysate samples were spiked with human recombinant TrxR1 (50 nM) and as expected, no difference in NADPH consumption (μmol/min/mg) in the GPX assay compared to control lysate samples without TrxR1 was observed (Fig. 1E; **filled vs. open bars)**. In agreement with Fig. 1C and D, the auranofin-treated samples (40 μM) showed strong suppression of the total GPX activity compared to the DMSO controls (Fig. 1E; **first two pairs of bars**), while the addition of TRi-1 (1 and 5 μM; TrxR1 IC_50_ = 12 nM), a more selective TrxR1 inhibitor than auranofin [65], [66], had no impact on NADPH consumption, consistent with an assay specific for the determination of GPX isoenzyme activities (Fig. 1E, **last two pairs of bars**). To confirm TrxR1 was active when spiked into HT1080 lysate, we tested its catalytic reduction of sodium selenite (100 μM), adapted from previously described TrxR1 activity assays [19], [34], and found that the enzyme retained its functionality in these conditions (Fig. 1F, **first pair of bars**). Spiked TrxR1 activity was also fully suppressed by auranofin (40 μM) and Tri-1 (1 and 5 μM) (Fig. 1F, **last three pairs of bars**), in contrast to the GPX activity that was only inhibited by auranofin and not by TRi-1 (Fig. 1E and F).

### 3.2 Probing the GPX assay using GPX1 and GPX2 knockdown cells

To assess the relative contributions of GPX1, GPX2 and GPX4 isoenzymes to the total cellular GPX activity in lysate, we next analyzed the assay performance using human colon cancer HT-29 cells with stable knockdown (KD) of either GPX1 or GPX2, as developed previously [54], [55]. Based on reports in the literature, as also discussed above, we hypothesized that GPX1 would be the predominant GPX isoenzyme in selenium-supplemented cells and account for the majority of cellular GPX activity. Consistent with this hypothesis, GPX1 KD cell lysates exhibited the lowest GPX activity (total GPX activity: 0.0032 ± 0.0006; GPX1/GPX2 activity: 0.0028 ± 0.0002 μmol/min/mg), which was approximately fourfold less than in the scrambled (SCR) wild type cell lysates (total GPX activity: 0.013 ± 0.0004; μmol/min/mg; GPX1/GPX2 activity: 0.012 ± 0.0002 μmol/min/mg) (Fig. 2A and **Supplement S1A**, and kinetic graphs 2C and 2D). The GPX2 KD lysates exhibited total GPX activity levels comparable to the SCR controls (total GPX activity: 0.012 ± 0.0006 μmol/min/mg; GPX1/GPX2 activity: 0.011 ± 0.0001 μmol/min/mg, n = 3), and GPX1 protein levels were similar between GPX2 KD and SCR lysates, whereas the GPX1 KD lysates showed no detectable GPX1 protein (Fig. 2A and B, **Supplement S1A and S1B-D** and kinetic graphs 2C and 2E). The low auranofin-inhibited activity in the GPX1 KD sample, assumed to be GPX2 activity, is consistent with the finding that GPX2 KD has a minimal effect on the total cellular GPX activity compared to GPX1. This finding also supports the notion that GPX1 is the isoenzyme primarily responsible for GSH-dependent CHP reduction in lysates of HT-29 cells, as determined by the auranofin-inhibited combined GPX1/GPX2 activities (Fig. 2A and **Supplement S1A**, **solid bars and dots)**. Therefore, in HT-29 cell lysates, the small and non-statistically significant portion of activity remaining after auranofin treatment (Fig. 2A and **Supplement S1A**, white bars and white dots) suggests that other GPX isoenzymes in the lysates – and/or some other unidentified GSH-dependent peroxide reduction activity – would contribute minimally to the total GPX activity relative to GPX1.

**Fig. 2:**
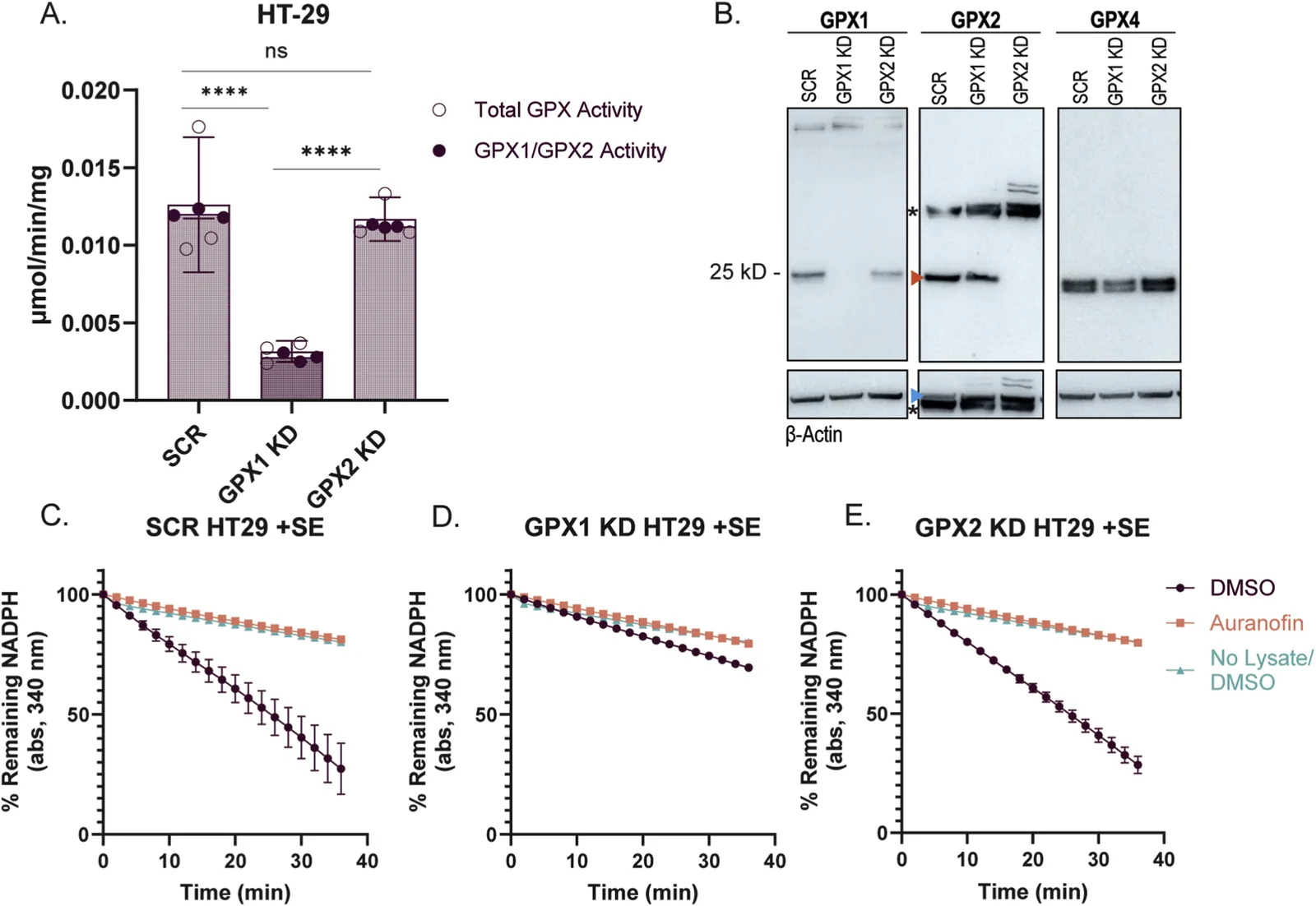
Total GPX and GPX1/GPX2 activity of GPX1 and GPX2 KD HT-29 cell lysate from selenium-supplemented culture conditions. **(A)** GPX1/GPX2 activity (solid purple bars and dots), corresponding to the auranofin-inhibited portion of the total activity, is measured as the rate of NADPH consumption per mg lysate (μmol NADPH/min/mg). This is superimposed on total GPX (white bars and dots) activity for each KD and scramble (SCR) cell lysate sample (50 μg/well), see **Supplement S1A** for a version of this graph with separated bars. n = 3 technical replicates per condition, ns = not significant, **** = p < 0.001. **(B)** Western blot immuno-detection of GPX1 (∼23 kDa), GPX2 (∼22 kDa), and GPX4 (20-22 kDa) in HT-29 SCR, GPX1 KD and GPX2 KD cell lysates. β-Actin (∼42 kDa) was used as a loading control, 10 μg of protein was loaded per lane. Blots were first probed for GPXs, then washed 3x and probed for β-Actin. A non-specific band (marked with *, ∼40 kDa) was observed in the GPX2 and corresponding β-Actin blot (orange arrowhead = GPX2, blue arrowhead = Actin). Intact whole blot images shown in **S1B-D**. **(C-E)** Kinetic graphs: % remaining NADPH over time (minutes) for HT-29 SCR, GPX1 KD and GPX2 KD cell lysates with auranofin or DMSO treatment, the decrease in absorbance over time (abs/min) was used to determine the rate of NADPH consumption μmol NADPH/min/mg in panel A. For all kinetic graphs, a no lysate control (teal) treated with DMSO shows background activity where slight NADPH consumption is observed in the absence of cell lysate.

### 3.3 Total GPX activity in cell lysates is selenium dependent and correlates with GPX1 protein levels across cancer cell lines

To ensure the lysate assay is generalizable to other cell lines, we evaluated its performance across four additional human cancer cell lines: HT1080 fibrosarcoma, A549 lung adenocarcinoma, MDA-MB-453 triple-negative breast cancer, and HEPG2 hepatocellular carcinoma. These cell lines were selected for their varied GPX1, GPX2 and GPX4 protein profiles, allowing us to assess whether isoenzyme abundance correlates with GPX activity. As expected [12], [13], [14], [18], the expression of all three Sec-containing GPX isoenzymes (GPX1, GPX2 and GPX4) was responsive to selenium supplementation in the cell culture medium. We detected minimal to no total GPX activity in all samples derived from cells grown without addition of selenium (Fig. 3A), which correlated with very low or undetectable protein levels of all three isoenzymes (GPX1, GPX2 and GPX4) in the corresponding immunoblots (Fig. 3B). The GPX1/GPX2 activity was again the primary contributor to the total cellular GPX activity, that is the reduction of CHP and coupled NADPH consumption by GR, across all selenium-supplemented cell lines, accounting for between 58% (A549) to 82% (HT1080) of the total GPX activity (Fig. 3A and kinetic graphs 3E-H). In selenium-supplemented conditions, the HEPG2 sample had the highest total GPX, GPX1/GPX2 and non-GPX1/GPX2 activities, while A549 had the lowest total GPX and GPX1/GPX2 activities (Fig. 3A). Notably, the GPX1/GPX2-specific activities correlated significantly with the GPX1 protein levels (integrated density of immunoblots in Fig. 3B) in the different cell lysates (Fig. 3C), whereas GPX2 protein levels did not significantly correlate with activity **(Supplement S2B and S2C)**. Together with the near-complete suppression of GPX activity observed in HT-29 GPX1 KD lysates (Fig. 2A), these results further support GPX1 as the primary contributor to the total GSH-dependent (and auranofin-inhibitable) CPH reduction capacity in the evaluated cell types.

**Fig. 3:**
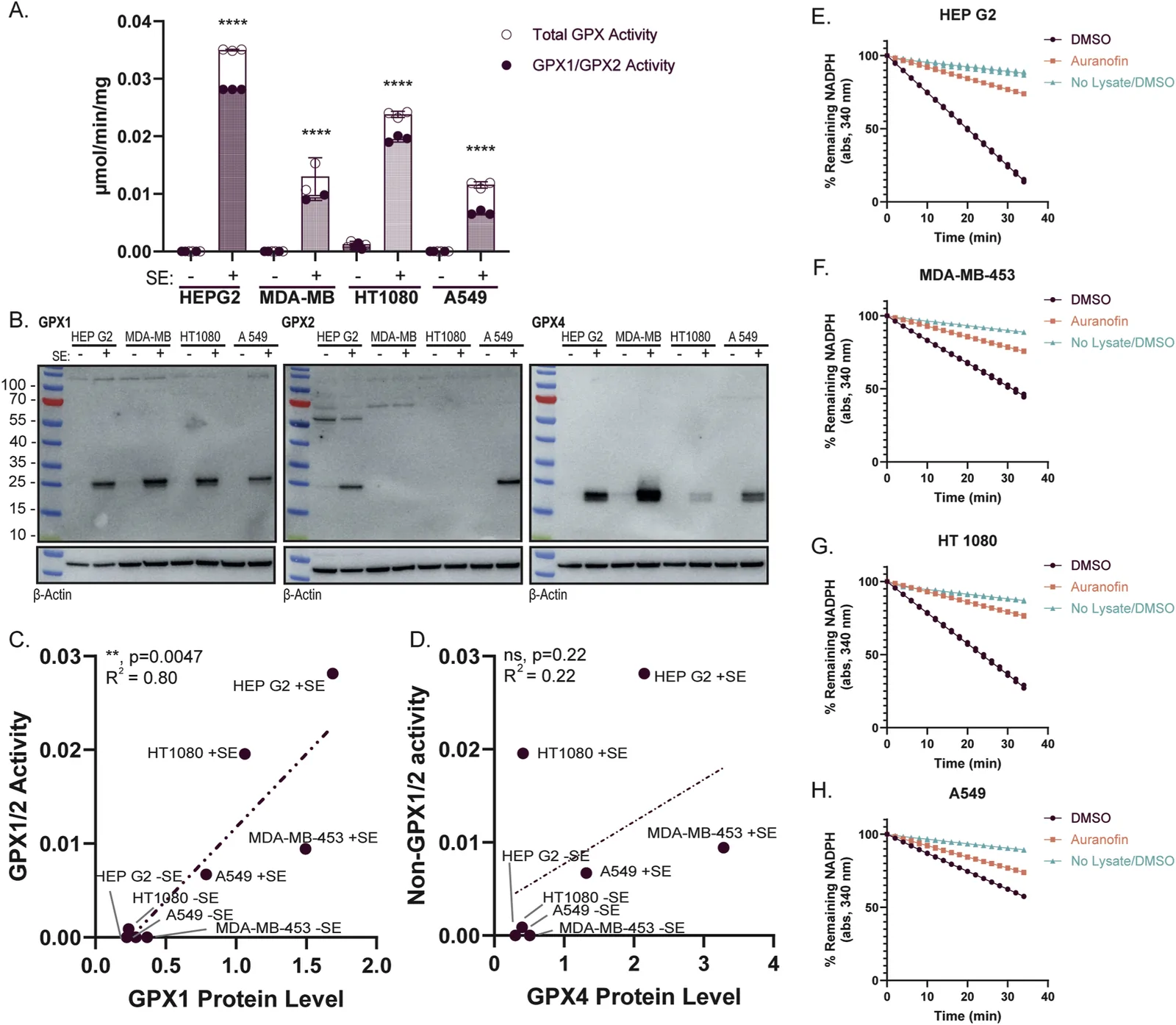
GPX activity and protein level profiles for lysates prepared from four human cancer cell lysates: HEPG2, MDA-MB-453 (MDA-MB), HT-1080, and A549 grown in selenium-supplemented (+SE) and non-supplemented (-SE) cultures. **(A)** GPX1/GPX2 activity (solid purple bars and dots), the rate of NADPH consumption per mg lysate protein (μmol NADPH/min/mg), was superimposed on total GPX (white bars and dots) activity per auranofin or DMSO-treated condition (n = 3), **** = p < 0.001 compares +SE samples to -SE samples for all cell types. **(B)** Western blot immuno-detection of GPX1 (∼23 kDa), GPX2 (∼22 kDa), and GPX4 (20-22 kDa). β-Actin (∼42 kDa) was used as a loading control, and 10 μg of protein was loaded per lane. A linear regression and correlation between **(C)** GPX1/GPX2 lysate activity and GPX1 protein level and **(D)** Non-GPX1/GPX2 activity and GPX4 protein levels. **3C and 3D** x axis: the integrated density of immunoblot bands normalized to Actin controls from **3B; 3C and 3D** y axis: GPX1/GPX2 activity from **3A. (E-H)** Percent remaining NADPH consumption over time (minutes) for + SE cell lysates **(3A)** treated with either auranofin (orange squares) or DMSO (purple circles). A no lysate control (teal triangles) treated with DMSO was included to show background NADPH consumption.

The remaining “non-GPX1/GPX2 activity”, not inhibited by auranofin in our assay, could include both GPX4-derived and additional GPX-like activities in these cell lysates. This accounted for between 20% (HT1080) and 42% (A549) of the total GPX activity, which was lower compared to that derived from GPX1/GPX2 (Fig. 3A and **Supplement S2A**). Despite notably higher GPX4 protein levels in the MDA-MB-453 samples, the non-GPX1/GPX2 activities were the lowest in those cells relative to the other cell lysates analyzed here (Fig. 3A and B and **Supplement S2A**). Further analysis showed that the non-GPX1/GPX2 activity did not correlate significantly with the GPX4 protein levels (Fig. 3D), suggesting that other enzymes contribute to the portion of GPX-like activity uninhibited by auranofin. Our results support previous findings that GPX4 activities in cell lysates, even from cells cultured in the presence of selenium and with relatively high GPX4 protein levels, are typically too low to be detected via measurement of GR-coupled NADPH consumption [53]. This prompted us to explore an alternative cell-based method for assessing GPX4 in cells, but first, we employed the GPX1/GPX2-specific assay to evaluate the effects of compounds presumed to target GPX isoenzymes in cell lysate.

### 3.4 Evaluation of total GPX and specific GPX1/GPX2 activity in lysate treated with GPX-targeting compounds

To assess the isoenzyme-specific inhibition of GPX activity in cell lysates by compounds of interest, we utilized the methods described above with the addition of a 30-min compound incubation (Fig. 4A, Step 3b), in contrast to the single 15-min auranofin incubation for the assays above, outlined in Table 1 and Fig. 1A, Step 3a. For this, we selected three GPX1/GPX2 inhibitors (auranofin, ceftibuten and omapatrilat) discovered in a biochemical screening campaign using recombinant GPX protein [19], along with RSL3, a ferroptosis-inducing compound that inhibits cellular GPX4 [27], [28], [67]. RSL3 induces ferroptosis at sub-micromolar concentrations, however it does not directly inhibit GPX4 at these concentrations [34], [53]. We treated HT1080 lysates with three different concentrations of each compound and assessed total GPX and GPX1/GPX2-specific activities (Fig. 4B). Among the GPX1/GPX2 inhibitors, omapatrilat strongly inhibited the total GPX activity and GPX1/GPX2-specific activity, followed by ceftibuten (Fig. 4B). Auranofin demonstrated weaker potency and an apparent increase in non-GPX1/GPX2 activity. These results demonstrate that the direct GPX1 inhibition observed for auranofin, omapatrilat and ceftibuten using pure enzyme assays [19] translates to GPX1/GPX2 inhibition in cell lysates. However, the potency of each compound differs between enzyme and lysate assays, suggesting that cellular context and/or off-target interactions influence their inhibitory activity.

**Fig. 4:**
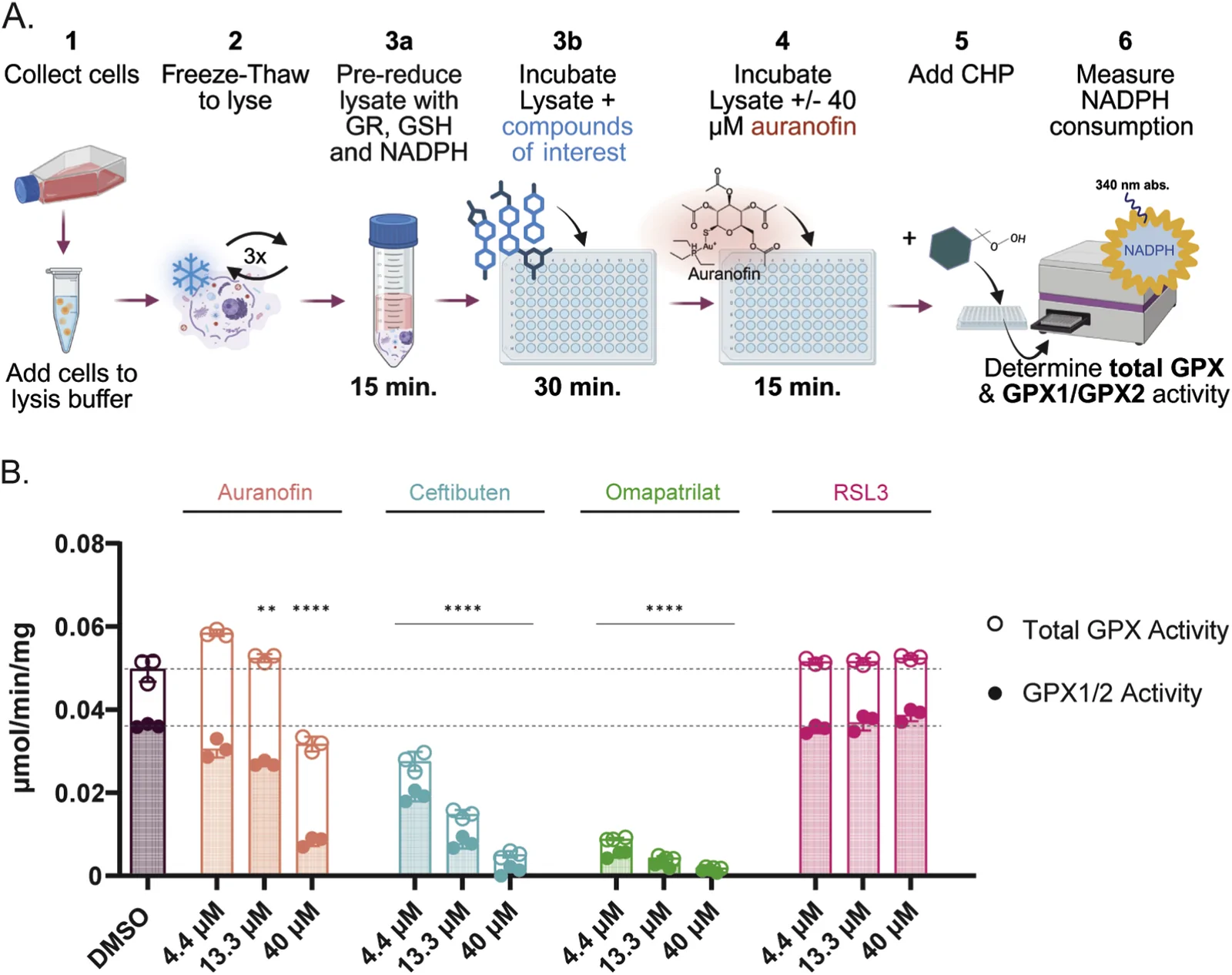
Assessing total GPX and GPX1/GPX2 activity in lysates treated with compounds of interest. **(A)** Schematic depicting the lysate assay protocol including the incubation with compounds of interest (Step 3b) prior to the 15-min auranofin incubation (Step 4) for determining GPX1/GPX2 lysate activity. Created with Biorender.com. **(B)** The inhibition of GPX1/GPX2 (solid bars) and total GPX activity (white bars) in HT1080 + SE crude cell lysate following a 30-min treatment (workflow described in **panel A**) with GPX1 biochemical inhibitors (auranofin, ceftibuten and omapatrilat), a ferroptosis inducer (GPX4-targeting compound), RSL3, or DMSO control. Dashed lines represent the DMSO control total GPX (0.045 ± 0.003 μmol NADPH/min/mg) and GPX1/GPX2 (0.036 ± 0.003 μmol NADPH/min/mg) HT-1080 lysate activity, **p = 0.02, ****p < 0.0001 compared to DMSO-treated lysate (n = 3).

In line with our previous findings that RSL3 does not inhibit GPX1, GPX2 or GPX4 in pure enzyme assays [34], here we also observed that in HT1080 cell lysates, RSL3 does not inhibit either total GPX or the GPX1/GPX2-specific lysate activities (Fig. 4B). These findings indicate that the effects of RSL3 on cellular GPX isoenzymes cannot be readily corroborated using the GPX lysate activity assay. As stated above, GPX4-specific activity cannot be measured using this approach, so we proceeded to establish an orthogonal cellular target engagement assay for the evaluation of compounds with potential GPX-targeting activity.

### 3.5 Development of GPX1-HiBiT and GPX4-HiBiT cell lines

Like Nakamura et al., we were also unable to measure significant endogenous GPX4 lysate activity using a GR-coupled spectrophotometric activity assay [53]. As an alternative assay that enables the evaluation of cellular target engagement by novel modulators, we developed intact-cell CETSA assays using HT1080 cells with CRISPR-Cas9-mediated insertion of a HiBiT peptide tag at the endogenous GPX1 or GPX4 loci (Fig. 5A). In these assays, compound-treated cells are heated at a defined temperature and time, and compound-induced changes in protein thermal stability are quantified by measuring the remaining soluble fraction of HiBiT-tagged GPX1 or GPX4. Following cell lysis, the HiBiT peptide tag complexes with a LgBiT subunit to form a functional NLuc luciferase that luminesces in the presence of an exogenous luciferase substrate in an ATP-independent manner. This signal is proportional to the amount of soluble HiBiT-tagged protein and provides a simple, quantitative approach to assessing compound target engagement in two distinct HT1080 cell populations [50].

**Fig. 5:**
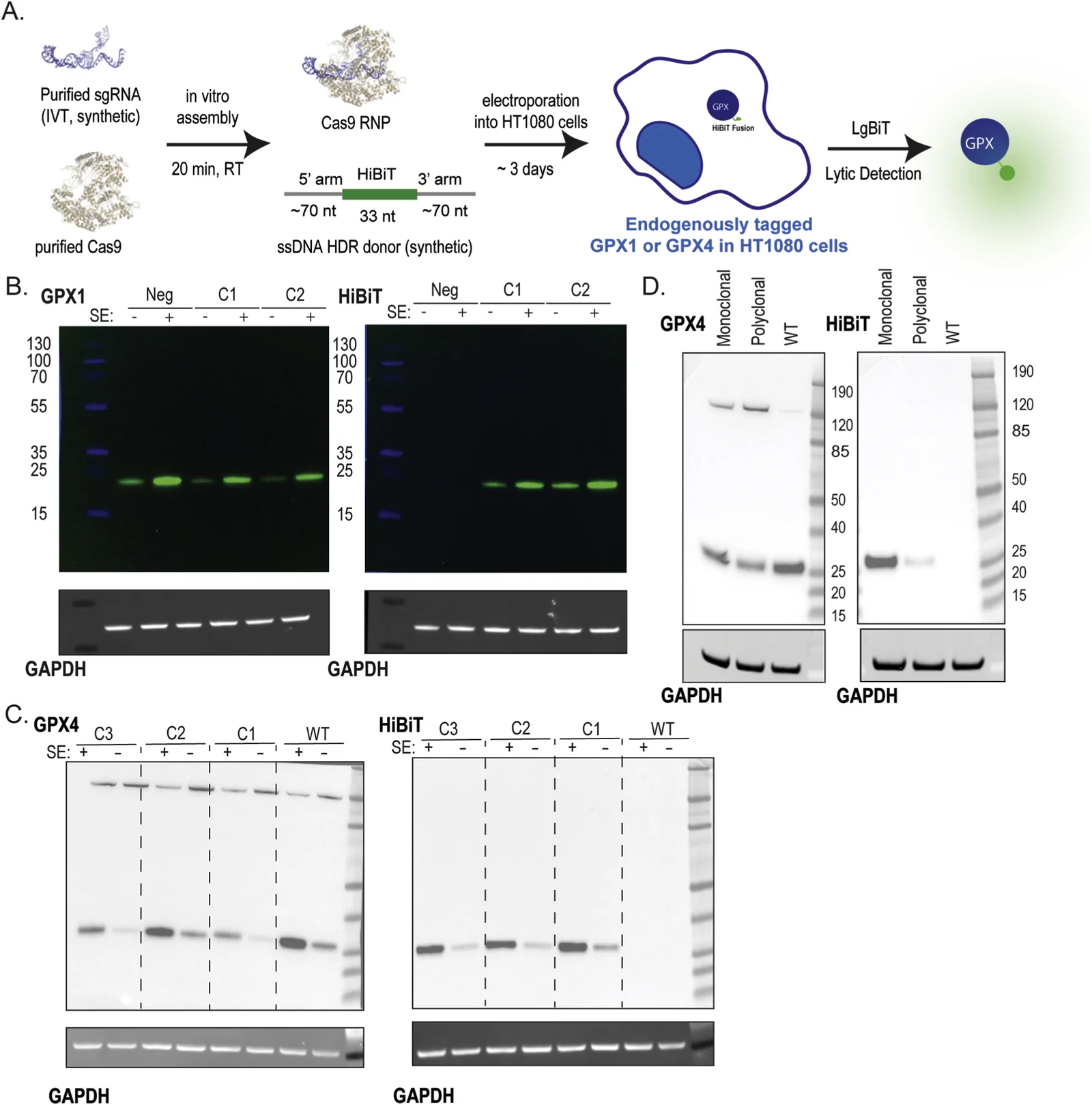
Generation and validation of HiBiT peptide tag insertion into GPX1 and GPX4 in HT1080 cells. **(A)** Schematic depicting HiBiT tag nucleofection procedure: first, the CRISPR Ribonucleoprotein (RNP) is assembled, next the RNP and Homology-Directed Repair (HDR) ssDNA donor template is electroporated into HT1080 cells. After 3 days of growth, NLuc signal (HiBiT-LgBiT complex) is detectable in lysed cells. Western blot immuno-detection of **(B)** GPX1 (∼23 kDa, left blot) and the GPX1-HiBiT tag (right blot). In lanes 1 and 2: HT1080 untagged negative control (no Cas9, Neg), lanes 3-6: the GPX1-HiBiT C-terminus tag inserted using two different gRNA sequences (C1, C2). **(C)** GPX4 (20-22 kDa, left blot) and the GPX4-HiBiT tag (right blot). In lanes 1-6: the GPX4-HiBiT C-terminus tag inserted using three gRNA sequences (C1, C2 and C3). Cells were grown either in selenium (SE) supplemented (+) or non-supplemented (−) conditions. **(D)** Immuno-detection of GPX4 and GPX4-HiBiT tag in monoclonal or polyclonal HiBiT-tagged, and WT (no HiBiT tag), HT1080 cell populations (supplemented with selenite).

In parallel experiments, we tested at least two single-guide RNAs (gRNA) targeting either the C- or N- termini of GPX1 or GPX4 in HT1080 cells. Only the C-terminally tagged constructs demonstrated significant NLuc luminescence in HT1080 for both the GPX1 and GPX4 (Fig. 5B and C, C1-C2/C3 for GPX1 and GPX4). Next, we verified successful HiBiT integration at either the GPX1 or GPX4 locus by immunoblotting, comparing each HiBiT-tagged population to WT HT1080 cells (Fig. 5B and C). Anti-HiBiT immunoblotting confirmed the presence of HiBiT-tagged protein at the expected molecular weight in GPX1-HiBiT and GPX4-HiBiT cells and demonstrated that the tagged proteins retained selenium-responsive expression (Fig. 5B and C). We selected the gRNA-directed HiBiT-tagged populations with the strongest signals for further analyses (GPX1: C2, GPX4: C1, Fig. 5B and C). To enhance assay sensitivity in the GPX4-HiBiT HT1080 population, which displayed a notably lower absolute NLuc signal compared to the GPX1-HiBiT HT1080 polyclonal cells, single-cell clone selection was performed. A clone with increased anti-HiBiT expression relative to the parent polyclonal population was selected for further experiments (Fig. 5D). Next, we confirmed the enzymatic functionality of the GPX1-and GPX4-HiBiT-tagged proteins using the lysate activity assay, recognizing that GPX4 activity can only be inferred from this assay, as discussed above. Total GPX and GPX1/GPX2 activities were slightly higher in lysates from the GPX4-HiBiT monoclonal cell line than in wild-type HT1080 cells **(Supplement S3A)**, likely reflecting clonal variation arising from single-cell selection. GPX1-HiBiT lysates exhibited GPX activities comparable to those of wild-type HT1080 cells **(Supplement S3A)**, indicating that HiBiT tagging did not impair detectable enzymatic activity.

Using the GPX4-HiBiT monoclonal and GPX1-HiBiT polyclonal HT1080 cells, we next established the optimal temperatures for the subsequent CETSA assays. We measured baseline GPX1-and GPX4-HiBiT signals at five increasing temperatures to create a temperature response plot corresponding to GPX1 or GPX4 protein denaturation (Fig. 6A). Based on those results, we selected 55°C for GPX4, with 30% GPX4-HiBiT signal remaining (Fig. 6A, **right** **bar graph**), and 60°C for GPX1, with 40% GPX1-HiBiT signal remaining (Fig. 6A, **left** **bar graph**) compared to 37°C control, for subsequent studies. We reasoned that assessing the signals at these respective temperatures would enable detection of both destabilizing (loss of luminescence) and stabilizing (gain of luminescence) effects of GPX1-HiBiT- or GPX4-HiBiT-targeting compounds, both of which are possible in the presence of a protein ligand [32], [51], [61].

**Fig. 6:**
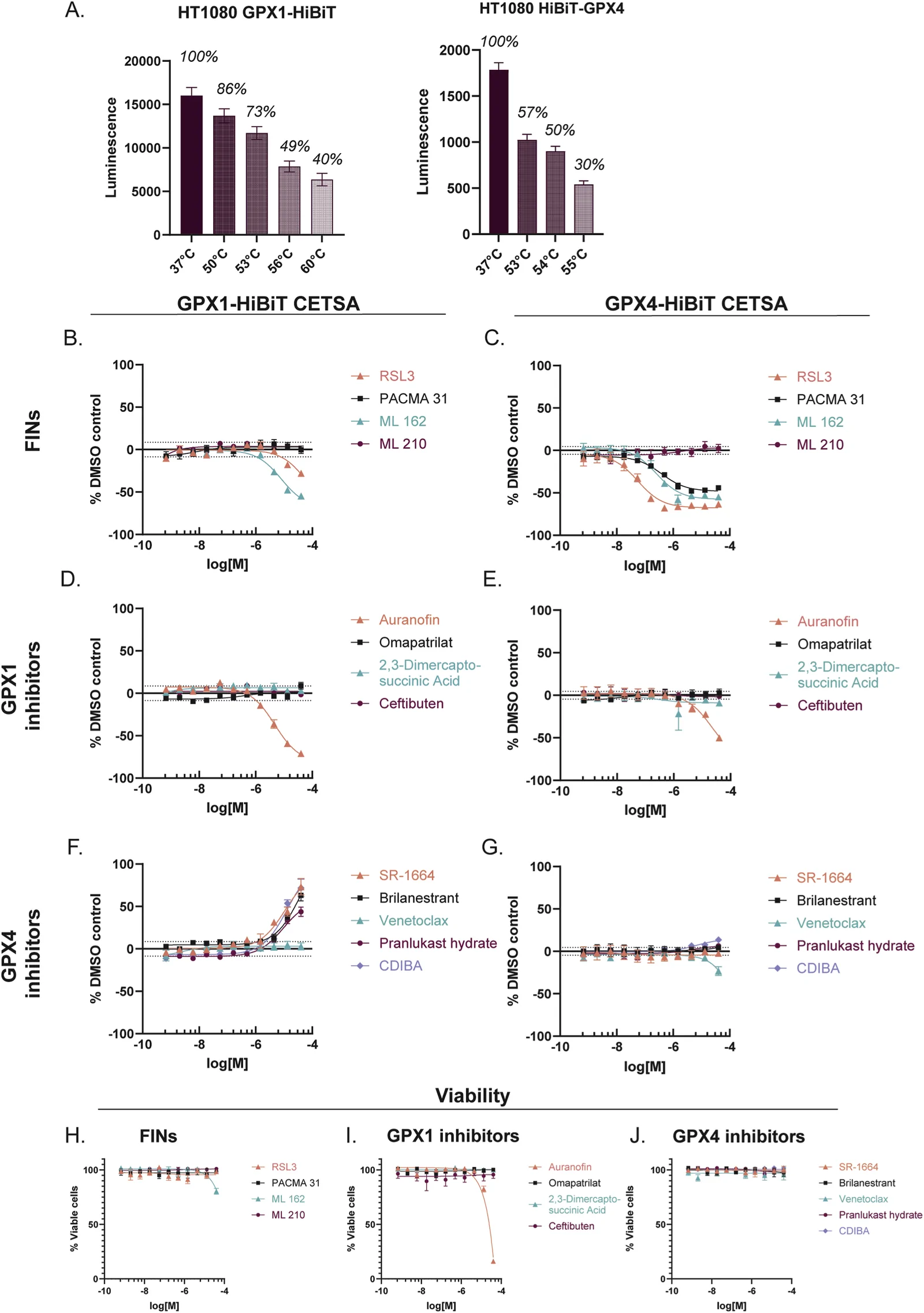
Target engagement of GPX biochemical inhibitors or ferroptosis inducers (FINs) with GPX1 and GPX4 in HiBiT-tagged HT1080 cells. **(A)** GPX1-HiBiT (left) and GPX4-HiBiT (right) HT1080 NLuc complex (HiBiT-LgBiT) at increasing temperatures, heated for 3.5 min. Values above bars (% GPX HiBiT-LgBiT luminescence) are normalized to 37°C samples. N = 96 per temperature. CETSA profiling of **(B and C)** FINs (cellular GPX4-targeting compounds), **(D and E)** recombinant GPX1 and **(F and G)** GPX4 inhibitors treated for 1 h on GPX1-HiBiT, polyclonal, (60°C) **(B, D and F)** and GPX4-HiBiT, monoclonal, (55°C) **(C, E and G)** HT1080 cells grown in selenium-supplemented conditions and heated for 3.5 min. Horizontal dashed lines in CETSA graphs represent±standard deviation of DMSO controls (N = 3). **(H, I and J)** Viability of HT1080 cells treated for 1 h with the compounds in panels **B-G** (N = 4)**.**

### 3.6 Evaluation of GPX lysate inhibition, GPX1-HiBiT and GPX4-HiBiT target engagement by putative GPX isoenzyme inhibitors

To evaluate cellular target engagement, we assessed a panel of compounds previously reported to inhibit GPX1 or GPX4 in pure enzyme assays [19], as well as presumed GPX4-targeting ferroptosis inducers (FINs), using intact, live-cell CETSA assays with a HiBiT-LgBiT lytic readout (Fig. 6, **Supplement S3** and Table 2). Notably, inhibition of cellular enzyme activity and effects on biophysical thermostability are not directly comparable readouts; a compound may theoretically bind an enzyme without inhibiting its enzymatic activity or inhibit an enzyme activity without producing detectable thermal stabilization or destabilization. Thus, the different assay readouts need to be interpreted accordingly.

**Table 2:**
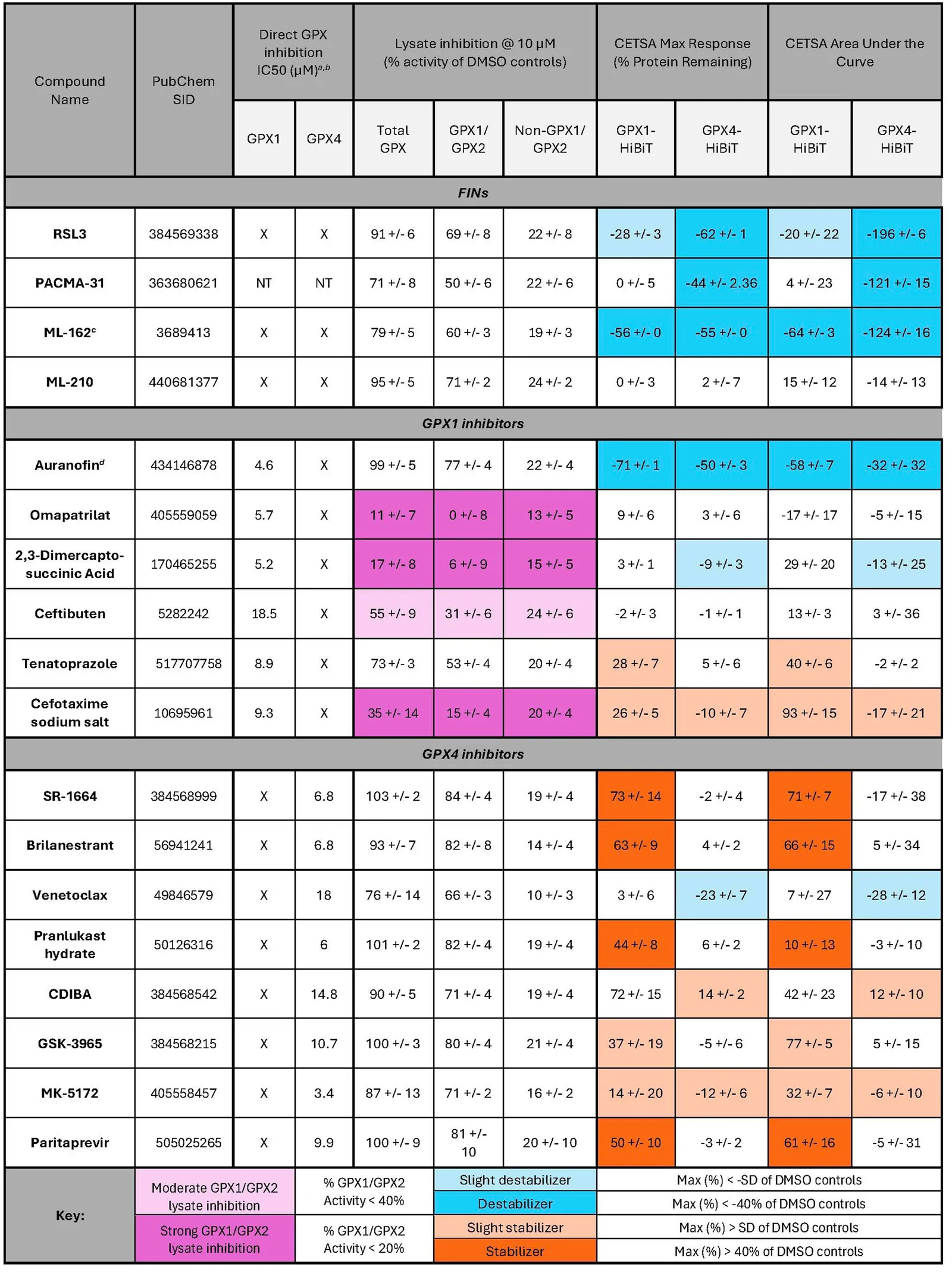
GPX isoenzyme lysate inhibition, GPX1-HiBiT and GPX4-HiBiT CETSA results for the evaluated FINs, GPX1 and GPX4 inhibitors in Fig. 6 and **Supplement S3C-F**. HT1080 cells (grown in selenium-supplemented medium) were treated for 30 min at 10 μM to determine inhibition of total GPX, GPX1/GPX2 and non-GPX1/GPX2 activities, reported as respective portions of the DMSO control total GPX activities (% Activity). See **Supplement S5** for NADPH consumption (μmol/min/mg) and corresponding bar graph. CETSA max response at the top concentration of compound is reported as % protein (either GPX1-HiBiT or GPX4-HiBiT) remaining compared to DMSO controls, and area under the curve (AUC) represents the integrated area between the concentration response curve and the x-axis in Fig. 6B–G **and S3C-F**. A larger deviation from zero represents a greater change in thermostability (see (*) in materials and methods for AUC calculation details).

^a^ Direct GPX inhibition IC50s determined with recombinant GPX1 or GPX4 in Cheff et al. [19], [34], except for PACMA-31 where the IC50 was calculated from activity assays in Supplement S4.

^b^ ML-162 shows slight interference in NLuc signal used in the GPX-HiBiT CETSA readout (Supplement S3B).

^c^ Auranofin is cytotoxic at top concentrations within 1 h of treatment applicable to GPX-HiBiT CETSA readouts (Fig. 6H–J).

X = inactive in the enzymatic activity assay.

NT = Not tested in the GPX enzymatic activity assays.

As GPX4 contributes little to the total GPX activity as measured in cell lysates using GSH-dependent CHP reduction (Fig. 2, Fig. 3), and because the mechanisms of small molecule target engagement with GPX4 may differ within the context of living cells compared to biochemical or lysate activity assays (Fig. 4B), the lack of RSL3-derived effects on GPX activities in lysates does not rule out engagement of either GPX1 or GPX4 by RSL3 in intact cells. Indeed, the CETSA assays with GPX1-HiBiT (Fig. 6B–Table 2) and GPX4-HiBiT (Fig. 6C–Table 2) demonstrated that RSL3 is a slight thermal destabilizer of GPX1, and a strong, dose-dependent destabilizer of GPX4 in lives cells (Fig. 6B and C, Table 2). We also evaluated the lesser-known FIN, PACMA-31 [68], which was first discovered as a protein disulfide isomerases (PDI) inhibitor [69], because unlike ML-162 and RSL3, it is the only FIN we have tested [34] that directly inhibits GPX4 in the enzymatic activity assay (IC_50_ of 1.3 μM) **(Supplement S4,** Table 2**)**. Like ML-162 and RLS3, PACMA-31 also directly inhibits TrxR1 (IC_50_ = 3 μM) [70]. All three FINs (RSL3 [67], ML-162 [28], [62] and PACMA-31), thermally destabilized GPX4 in a clear dose-dependent manner (Fig. 6C–Table 2), demonstrating target engagement of GPX4 in the intact cellular context. This aligns well with Eaton et al. who tested ML-162, RSL3 and ML-210 on cells at a single concentration in CETSA, using Western blots to monitor protein levels at increasing temperatures [32]. In that study, ML-210 was reported as a thermo-stabilizer of GPX4 [32], however here we observed only a very slight stabilization of GPX4-HiBiT (Fig. 6C). We performed a counter assay to evaluate compound interference with NLuc complexing and/or luciferase activity and found that of the compounds tested here, only ML-162 demonstrated some NLuc interference (**Supplementary text,** Supplement Table 1 and **Supplement S3B**).

The HiBiT CETSA assays developed here use an acoustic compound transfer to 384-well plates and liquid handling instrumentation, enabling high-throughput testing of small molecules. This allowed us to test several previously reported GPX1/GPX2 and GPX4 biochemical inhibitors [19], in addition to the previously discussed FINs, in 11-point concentration-response format in both our GPX1-HiBiT and GPX4-HiBiT CETSA assays (Fig. 6B–G, Supplement S3C-F). Compared to thermostabilizing compounds, resulting in a higher NLuc luminescence than DMSO controls, destabilization of GPX1 or GPX4 (loss in NLuc luminescence) could also be due to a loss of viable cells, if not adjusted for the numbers of viable cells in the measured samples. For all compounds tested, we thus determined their cytotoxic effects within the time frame of the CETSA assay (Fig. 6H–J, Supplement S3G-H). After only 1 h of treatment, we found that no FINs (Fig. 6H) and a minority of the GPX inhibitors (Fig. 6I and J, Supplement S3G-H) were cytotoxic to the analyzed HT1080 cells, indicating that the observed decreased NLuc signals, with the exception of ML-162 which was found to interfere with NLuc luminescence **(Supplement S3B),** should indeed correspond to protein destabilization due to either indirect effects or direct binding of the molecules to the GPX1-HiBiT or GPX4-HiBiT proteins in the cells. Of the destabilizing compounds tested, auranofin (Fig. 6I) was the only cytotoxic compound within the observed treatment time, which may explain the corresponding loss in NLuc luminescence in both CETSA assays (Fig. 6D and E), rather than, at least solely, reflecting target engagement.

Most of the compounds tested have previously only been reported as inhibitors of recombinant GPX1 or GPX4 enzymes [19] and have not yet been evaluated for isoenzyme-specific target engagement in cells or GPX lysate inhibition. We first profiled these compounds in the auranofin-enabled GPX isoenzyme-specific lysate assay, assessing their effects on total GPX, GPX1/GPX2 and non-GPX1/GPX2 activity in HT1080 selenium-supplemented lysate at 10 μM (Table 2, Supplement S5). The FINs showed some inhibition of GPX activity in cell lysate, particularly PACMA-31 and ML-162, however the strongest total GPX and GPX1/GPX2 inhibitors were indeed compounds which directly inhibited the pure GPX1 isoenzyme. As observed in Fig. 4B, auranofin was the weakest GPX1 inhibitor in cell lysate compared to the five other GPX1 inhibitors, clearly requiring a high concentration of compound to inhibit within this context. The GPX4 inhibitors demonstrated little to no effect on GPX1/GPX2 lysate activity, aligning with their isoenzyme direct inhibition profile reported previously [19], and some, such as venetoclax, exhibited lower non-GPX1/GPX2 activity compared to GPX1 inhibitors and FINs, suggesting GPX4 lysate activity may be suppressed, though again, this cannot reliably measured in this assay (Table 2, Supplement S5).

Following lysate activity profiling, we evaluated the same set of compounds for target engagement in the GPX1-HiBiT and GPX4-HiBiT CETSA assays. Of the previously identified direct inhibitors of GPX1, besides auranofin's destabilizing or likely cytotoxic effects, only tenatoprazole and cefotaxime produced a slight stabilizing effect on GPX1-HiBiT, indicating cellular GPX1 target engagement (Table 2, Supplement S3C), and had no thermal stability effects on GPX4-HiBiT (Table 2, Supplement S3D). Interestingly, the biochemical inhibition profile of the GPX4 inhibitors, SR-1664, brilanestrant and pranlukast, did not correspond to CETSA target engagement of GPX4. These three compounds, as well as GSK-3965 and paritaprevir, instead strongly stabilized GPX1-HiBiT at the top three concentrations, with a max response >40% of the DMSO controls (Fig. 6F, Supplement S3E, Table 2) and had no thermal effects on GPX4-HiBiT (Fig. 6G, Supplement S3F, Table 2). Though smaller in magnitude compared to the GPX1 stabilization observed, CDIBA stabilized GPX4-HiBiT at the three highest concentrations, while venetoclax was a slight destabilizer (Fig. 6G, Supplement S3F, Table 2). Though target engagement does not equate to inhibition, these inhibitors were previously found to be rather weak direct GPX4 inhibitors, with IC50s in the low micromolar range [19], and therefore the limited effects on GPX4 thermostability are not surprising. However, these compounds were also previously found not to inhibit recombinant GPX1 activity, and were considered selective GPX4 biochemical inhibitors, but here we observed a strong stabilization of GPX1-HiBiT by at least three of these compounds in HT1080 cells and slight GPX1/GPX2 HT1080 lysate inhibition at 10 μM. Together, these findings underscore the value of combining biochemical, lysate and intact-cell approaches for the comprehensive evaluation of GPX1/GPX2- and GPX4-targeting compounds.

## 4 Discussion

GPX1, GPX2 and GPX4 have received significant attention for their potential roles in cancer development, regulation of cellular redox balances and, for GPX4, control of ferroptosis. Despite this interest, there are no widely accessible or reliable methods to measure isoenzyme-specific GPX activities in cell lysates. This has limited the field's ability to interrogate the relative peroxide-reduction capacities of these enzymes in cells and to evaluate new pharmacologic modulators of their respective activities. The two isoenzyme-specific approaches developed here provide complementary tools for investigating the roles of GPX isoenzymes in cells. In the present study, these approaches have already yielded several new insights into GPX isoenzyme activity and target engagement.

Our results with cell lysates made from HT-29 cells with a stable knockdown of either GPX1 or GPX2 demonstrated that GPX1 accounts for the vast majority of the total GPX activity in these cells, consistent with earlier predictions [9]. We further validated that finding, and the selenium dependency of the total GPX activity, across four human cancer cell lines of different origin, selected based on differential GPX1 and GPX4 expression patterns (Human Protein Atlas; www.proteinatlas.org) and protein levels as assessed here by Western blots. Notably, although GPX4 protein expression varied across cell lines, the GPX1/GPX2-specific activity consistently dominated the total cellular GPX activity, and the GPX4 protein levels did not correlate with the residual (non-GPX1/GPX2) activities. GPX1's major contribution to total cellular GPX activity as found here aligns well with a previous study by Koeberle et al. finding that GPX2 knockdown in HT-29 cells suppressed the total GPX activity levels to ∼76% of controls, as compared to ∼36% upon GPX1 knockdown [55]. The results from our GPX1/GPX2-specifc lysate assay also highlight that the well-documented selenium hierarchy [3], [12], [13], [14], [18] does not correspond to relative activity between the isoenzymes in cell lysate in selenium-supplemented conditions. Because GPX1 is sensitive to selenium availability, the relative contributions to total GPX activity likely differ in non-supplemented systems; however, this could not be evaluated due to insufficient total GPX activity in selenium-poor lysates.

Our GPX1/GPX2-specific activity assay results agree with a previous report on the development of a GPX4-specific assay by Nakamura et al. which utilized GPX4-specific lipid substrates [53]. In their study, they also reported on the relatively low activity of GPX4 in cell lysates [53]. Mirroring our approach for the specific detection of GPX1/GPX2 in lysate, we also attempted to define GPX4 activities using GPX4-specific pharmacological inhibitors but were for several reasons unsuccessful. First, the effort is limited by the present lack of potent, direct GPX4 inhibitors that could serve as suitable tool compounds [19], [34], and second, the GPX4 activity in cell lysate appears too low to be reliably detected indirectly through its pharmacological inhibition. FINs, such as RSL3, are widely known as GPX4 inhibitors, however RSL3 does not directly inhibit recombinant GPX4 unless very high concentrations are used [34], and we observed no inhibitory effect on total GPX activity in cell lysates. However, evidently the cellular context may provide an adaptor protein, such as 14-3-3ε, which enables GPX4 inhibition [71], as suggested by Vučković et al.; even in the presence of cytosolic extracts, RSL3 lacked sufficient potency necessary for the development of a GPX4-specific lysate activity assay [71]. Nakamura et al. attempted setting up a GPX4-specific assay for cell lysates based upon GSH-dependent reduction of the GPX4-specific substrate, phosphatidylcholine hydroperoxide (PCOOH), and reported a low signal to background ratio and only marginal differences in NADPH consumption between WT and GPX4 KO A375 and HT1080 cells [53]. They attributed this finding, in part, to high background NADPH consumption by other GSH-dependent oxidoreductases [53]. The residual non-GPX1/GPX2 lysate activity we detected in our assays could indeed be attributed to other GPX isoenzymes, or possibly other GSH-consuming enzymes capable of reducing CHP, such as glutathione S-transferases (GST) [72], [73] or PRDX6, which relies on both GSH and πGST for its peroxide reduction activity [74].

In a previous study, Stolwijk et al. proposed GPX4-and GPX1-specific assays using stepwise addition of PCOOH and H_2_O_2_ to cell lysates [56]. In their study, overexpression of GPX4 in H292 cells increased the GPX4 activity, as expected, while the GPX1 activity remained consistent [56], clearly demonstrating the specific detection of GPX4 using PCOOH as substrate. However, they too described a remarkably low PCOOH-coupled GPX4 turnover rate, accounting for only about 2.4% of the H_2_O_2_-coupled total GPX activity in lysates from HepG2 cells, known for high GPX4 expression levels. Because H_2_O_2_ is a substrate utilized by all GPX isoenzymes, including GPX4, when used in GSH-coupled assays it will detect all GSH-dependent peroxide activity, however still likely to primarily be contributed by GPX1, at least under selenium supplemented conditions. Instead of H_2_O_2_, we selected CHP as the ideal substrate for our assay as it is stable, not degraded by catalases [45], and is rapidly reduced by GPXs to provide a substantial signal-to- background ratio. With the goal of making our assay more widely accessible, we chose not to use PCOOH for GPX4 activity detection because it is a challenging lipid peroxide to produce, is unstable, and, as the two prior studies show [53], [56], results in a catalytic rate that is often indistinguishable from background in crude cell lysates.

The thioredoxin system, governed by selenoprotein thioredoxin reductases, is the other major mammalian selenium-dependent reductive pathway overlapping with the GSH-dependent pathways in reductive redox balance regulation [8]. If paired with specific thioredoxin (Trx)/TrxR1 activity assays for cell lysates, developed previously [75], the GPX isoenzyme assay described here should enable studies which probe the interactions and relationship of the two enzyme systems. Importantly, here we ruled out the possibility that TrxR1-derived NADPH consumption could confound the results of our novel assay, considering that the GPX1/GPX2-specific inhibitor for this assay, auranofin, is several orders of magnitude more potent at inhibiting TrxR1 [66]. Nonetheless, as the addition of recombinant TrxR1 enzyme (>90% selenium content) [57] or the TrxR1 specific inhibitor, TRi-1, did not impact on the assay readout, we are confident that the presence of TrxR1 in a lysate does not distort the GPX1/GPX2 activity measurements.

Our results show that at lower concentrations, omapatrilat and ceftibuten (4.4 μM and 13.3 μM), as well as cefoxitame (10 μM), were more potent GPX1/GPX2 inhibitors than auranofin when added to HT1080 lysate. However, only auranofin destabilized GPX1-HiBiT and GPX4-HiBiT in our CETSA assay, which despite its cytotoxic effects, is consistent with GPX1 target engagement. Omapatrilat and ceftibuten had no effect on either HiBiT signal, while cefoxitame slightly stabilized both proteins, highlighting that inhibition measured in lysates does not necessarily correspond to detectable cellular target engagement. Less efficient GPX1 inhibition by auranofin in lysates, and an apparent increase in non-GPX1/GPX2 activity, could relate to our previously reported observation that GPX1 inhibition by auranofin can be suppressed or reverted by GSH [76], this confounding factor was circumvented in our lysate activity protocol using 40 μM auranofin and a shorter 15 min incubation with cell lysate before addition of substrate. It is important to acknowledge that all four of these molecules, as well as most of the compounds assessed here, have other primary mechanisms of action in cells. Auranofin's promiscuity with many other targets within cell lysate may thereby leave less free molecules available to bind lower-affinity targets. Omapatrilat is a hypertensive agent while ceftibuten is an antibiotic, and it could be considered that targeting of either GPX1 or GPX2 may mediate some of the (side) effects of these compounds in humans. A logical extension of the current approach would be to evaluate lysates prepared from compound-treated intact cells rather than treating lysates directly. This strategy would provide a more physiologically relevant assessment of GPX1/GPX2 inhibition by irreversible inhibitors and, following compound washout prior to lysis, would also permit evaluation of downstream changes in GPX protein expression, including those mediated by NRF2 activation.

The new high throughput GPX4-HiBiT CETSA assay is better suited for characterizing cellular GPX4 modulators compared to a GPX4 activity assay using lysates. As we are interested in GPX1 and GPX4 isoenzyme selectivity, we also developed the GPX1-HiBiT assay for parallel target engagement assessment of small molecule binders. Our results showed that the FINs (RSL3, ML162 and PACMA-31) target and destabilize GPX4-HiBiT in the cellular context, suggesting either direct or indirect targeting of the enzyme. This result is consistent with prior reports on RSL3 and ML-162 suggesting indirect targeting of GPX4 in cells [32], while validating the use of the GPX4-HiBiT construct for this type of readout. Another FIN, PACMA-31, which does directly inhibit recombinant GPX4, also targeted and destabilized GPX4 in the CETSA assay, demonstrating that it too either directly or indirectly targets GPX4 in cells. CETSA studies have shown that changes in cellular redox state can indirectly alter the thermal stability of select proteins [77]. Given that the compounds tested here have additional known cellular targets and may perturb cellular redox homeostasis, we cannot exclude the possibility that the observed thermal shifts of GPX1-or GPX4-HiBiT reflect indirect redox-dependent effects rather than direct target engagement.

Conversely, the majority of the direct GPX1/GPX2 and GPX4 biochemical inhibitors [19], [34] tested here showed little to no effect on GPX4-HiBiT thermostability, while several GPX4 inhibitors rather firmly stabilized the GPX1-HiBiT construct and demonstrated some GPX1/GPX2 inhibition in HT1080 cell lysates, suggesting that GPX1 is targeted, and perhaps more accessible than GPX4, in cells. Shared binding affinities for GPX1 and GPX4 is unsurprising as they are both GSH-dependent Trx-fold peroxidases which rely on the Sec for their catalytic activity, however, their active site binding pockets differ as GPX4's shallow, more hydrophobic active site is designed to be more accessible for complex lipid hydroperoxide substrates [2], [78].

Our results demonstrate that for GPX1 and GPX4, biochemical inhibition of recombinant enzyme does not consistently correlate with cellular engagement of the enzyme, exemplifying the insights enabled by these two complementary assays described here. This discrepancy may be due to differences in compound permeability, stability, or specific aspects of cellular context, such as GPX4's localization or lipid peroxide-reduction function in cells. For the latter, it should be noted that a deleterious human mutation of GPX4, R152H, leading to Sedaghatian-type Spondylometaphyseal Dysplasia (SSMD), results in GPX4 with retained enzymatic activity in a biochemical assay context, but a distorted ability to bind to cardiolipin and anchor itself within the cell membranes in a biologically relevant setting [24], [25]. These findings suggest that the GPX4-HiBiT thermostability assay, and possibly other biophysical cell-based methods, may be better suited for assessments of probes or conditions modulating GPX4 functions, rather than direct activity assays for this enzyme.

## 5 Conclusions

We have presented the development and validation of a novel GPX1/GPX2 isoenzyme-specific lysate activity assay alongside a GPX1 and GPX4-HiBiT CETSA platform. Together these two methods facilitate mechanistic studies of these GPX isoenzymes in a cellular context. The CETSA assays provide a solution to assessing cellular GPX4 with a scalable, high-throughput approach, that can be used to support the discovery and optimization of novel GPX4-targeting probes. The GPX1/GPX2 lysate assay extends beyond existing non-specific GPX activity assays by offering facile isoenzyme-resolved functional readouts.

Our validation studies substantiated the notion that GPX1 activity typically predominates the total GPX activity in lysates from selenium-supplemented cells, at least for the cell types studied herein, highlighting the importance of isoenzyme-specific measurements. The evaluation of established GPX1 and GPX4 inhibitors revealed their target engagement profile in a cellular context and illustrated the robustness of the CETSA-based assay. Collectively, these two orthogonal, cell-based approaches provide a robust and translationally relevant framework for interrogating GPX1/GPX2 and GPX4 biology as pharmaceutical targets, currently mostly studied in the context of cancer therapy. The two methods expand the experimental toolkit for therapeutic discovery and offer new opportunities to dissect the roles of these specific GPX isoenzymes in redox biology.

## CRediT authorship contribution statement

**Madeleine S. Barrett:** Writing – original draft, Visualization, Methodology, Investigation, Formal analysis, Data curation, Conceptualization. **Jonathan H. Shrimp:** Writing – review & editing, Validation, Methodology, Investigation, Formal analysis. **Alina Löser:** Writing – review & editing, Validation, Resources. **Dorian M. Cheff:** Writing – review & editing, Supervision, Methodology, Conceptualization. **Qing Cheng:** Writing – review & editing, Resources, Methodology. **Anna P. Kipp:** Writing – review & editing, Supervision, Resources, Project administration, Methodology, Funding acquisition, Conceptualization. **Matthew D. Hall:** Writing – review & editing, Supervision, Resources, Project administration, Methodology, Funding acquisition, Conceptualization. **Elias S.J. Arnér:** Writing – review & editing, Supervision, Resources, Project administration, Methodology, Funding acquisition, Conceptualization.

## Ethics declaration

The authors have NOT obtained informed consent from participants or their legal representatives. No specific patient material, individual patients or other participants were included in this study, thus not requiring any patient consent.

This study was performed in compliance with relevant laws, regulatory frameworks and guidelines where the research took place. Ethics committee approval was not required under relevant laws and institutional guidelines. Only recombinant proteins and commercial cell lines of human origin were used in this study, not requiring ethical permits.

## Funding

This work was partially supported by the intramural research programs of the National Center for Advancing Translational Sciences, National Institutes of Health. MSB was supported through a joint NIH-Karolinska Insitutet graduate training program. ESJA acknowledges funding from Karolinska Institutet, The Swedish Cancer Society (24 3482 Pj), and The Hungarian National Research, Development and Innovation Office (NKFIH), under the National Laboratories Program (National Tumor Biology Laboratory (2022-2.1.1-NL-2022-00010)), the Hungarian Thematic Excellence Program (under project TKP2021-EGA-44) and Project Grant K 146277.

## Declaration of competing interest

ESJA and QC are shareholders in Selenozyme AB, a company selling recombinant selenoproteins.

## Data Availability

Data will be made available on request.
