# Supplementary material for: Isoenzyme-specific determination of glutathione peroxidase activity in cell lysates and GPX1/GPX4-HiBiT cellular target engagement
Source: Redox Biol. 2026 Oct 21;98:104445. doi: 10.1016/j.redox.2026.104445 (PMC13734959; doi:10.1016/j.redox.2026.104445)
Supplement: Supplementary file 1 [file mmc1.pdf]

SUPPLEMENTARY MATERIAL

Isoenzyme-specific determination of glutathione peroxidase activity in cell lysates and GPX1/GPX4-HiBiT cellular target engagement

Madeleine S. Barrett[a,b], Jonathan H. Shrimp[a], Alina Löser[c], Dorian M. Cheff[b,d], Qing Cheng[b], Anna P. Kipp[c], Matthew D. Hall[a], Elias S.J. Arnér[b,e]

[a]*Division of Preclinical Innovation, National Center for Advancing Translational Sciences, National Institutes of Health, 9800 Medical Center Dr., Rockville, MD, USA 20850*

[b]*Division of Biochemistry, Department of Medical Biochemistry and Biophysics, Karolinska Institutet, SE-171 77 Stockholm, Sweden*

[c]*Department of Nutritional Physiology, Institute of Nutritional Sciences, Friedrich Schiller University Jena, Dornburger Str. 24, 07743 Jena, Germany*

[d] *Assays, Profiling, and Cell Sciences, Discovery Sciences, BioPharmaceuticals R&D, AstraZeneca, Pepparedsleden 1, 431 83 Mölndal, Sweden*

[e]*Department of Selenoprotein Research and the National Tumor Biology Laboratory, National Institute of Oncology, 7 Ráth György utca, 1122 Budapest, Hungary*

Supplementary Methods:

Single stranded guide RNA (gRNA) sequences:

GPX1

C1 (20-mer): AGGGGCGCCCTAGGCACAGC

GPX4

C2 (20-mer): ACTTGTGGAGCTAGAAATAG

C3 (20-mer): CTTGTGGAGCTAGAAATAGT

Supplementary text:

Assessment of GPX1- and GPX4-HiBiT NLuc readout interference

As a counter assay to our HiBiT-LgBiT CETSA assays for GPX1-HiBiT and GPX4-HiBiT isoenzyme target engagement, we investigated whether our compounds interfered with the NLuc luciferase activity. We treated GPX1-HiBiT HT1080 cells with our selection of compounds and immediately added the lytic buffer, LgBiT and substrate, as part of our CETSA protocol, to determine if a loss in signal occurred due to NLuc interference **(Supplement Table 1).** Those which thermally destabilized GPX-HiBiT, and thus showed a decrease in NLuc signal, are shown in **Supplement 3B**. Of all 18 compounds tested here, only ML-162 decreased NLuc signal by a significant margin at the top concentration of 26.6 µM (65.0 ± 2.5 % luminescence of the DMSO control) **(Supplement Table 1, Supplement 3B)**, suggesting that this small molecule interferes with the NLuc complex formation or inhibits its activity within the CETSA protocol's HiBiT/LgBiT/substrate incubation window (10 min). Therefore, another readout, such as western blot protein detection, would be necessary to determine ML-162 target engagement with GPX1 in cells, such as the approach in the Eaton *et al.* study, which also confirms cellular GPX4 target engagement in a low-throughput setting.

Supplementary Table 1. GPX-HiBiT NLuc interference max response (% NLuc inhibition compared to DMSO controls). DMSO controls CV = 3.3%, N=2.

Compound Name	GPX1-HiBiT-LgBiT NLuc activity (% DMSO) @ 26.6 µM
**RSL3**	93 +/- 0
**PACMA-31**	102 +/- 3
**ML-162**	65 +/- 4
**ML-210**	102 +/- 1
**Auranofin**[d]	99 +/- 6
**Omapatrilat**	105 +/- 2
**2,3-Dimercapto-succinic Acid**	102 +/- 2
**Ceftibuten**	99 +/- 4
**Tenatoprazole**	95 +/- 7
**Cefotaxime sodium salt**	92 +/- 8
**SR-1664**	99 +/- 5
**Brilanestrant**	103 +/- 2
**Venetoclax**	95 +/- 4
**Pranlukast hydrate**	100 +/- 5
**CDIBA**	103 +/- 0
**GSK-3965**	94 +/- 5
**MK-5172**	101 +/- 1
**Paritaprevir**	99 +/- 2

Supplementary Figures:

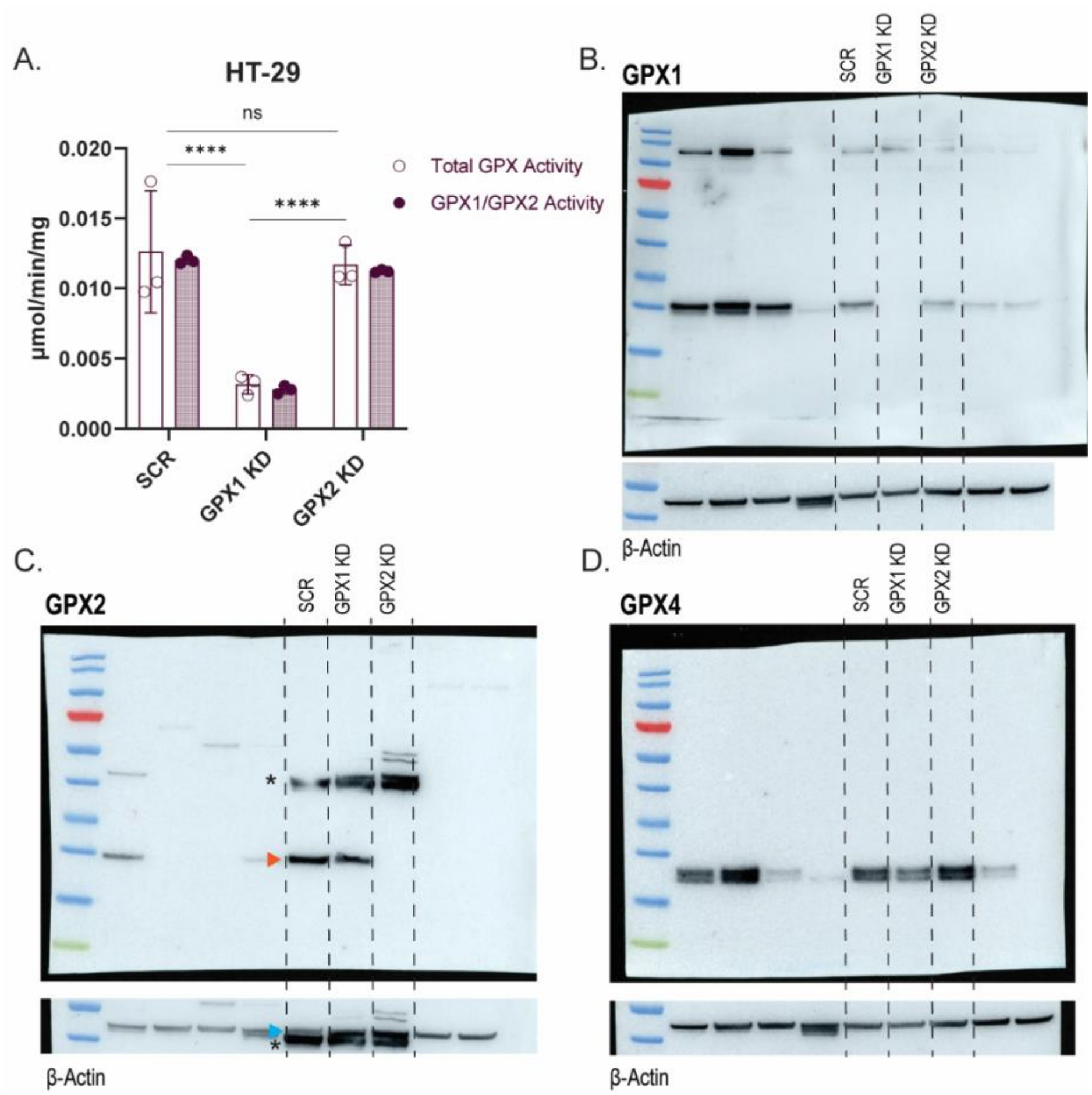

Supplementary Figure 1. (A) Main text **Figure 2A** graph shown here with separated bars for total GPX activity (open dots and bars) and the auranofin-inhibited, GPX1/GPX2 Activity (purple dots and bars) in HT-29 lysates. **(B-D)** Intact immunoblot images of **Figure 2B**, detection of GPX1, GPX2 and GPX4 in HT-29 SCR, GPX1 KD and GPX2 KD cell lysates. β-Actin (~42 kDa) was used as a loading control, at a slightly higher molecular weight than non-specific antibody labeling at ~40 kDa (marked with *) in the GPX2 KD HT-29 lysates (middle blot, GPX2 = orange arrow, Actin = blue arrow). 10 µg of samples loaded per lane, the unlabeled lanes contain samples for a separate, unrelated study.

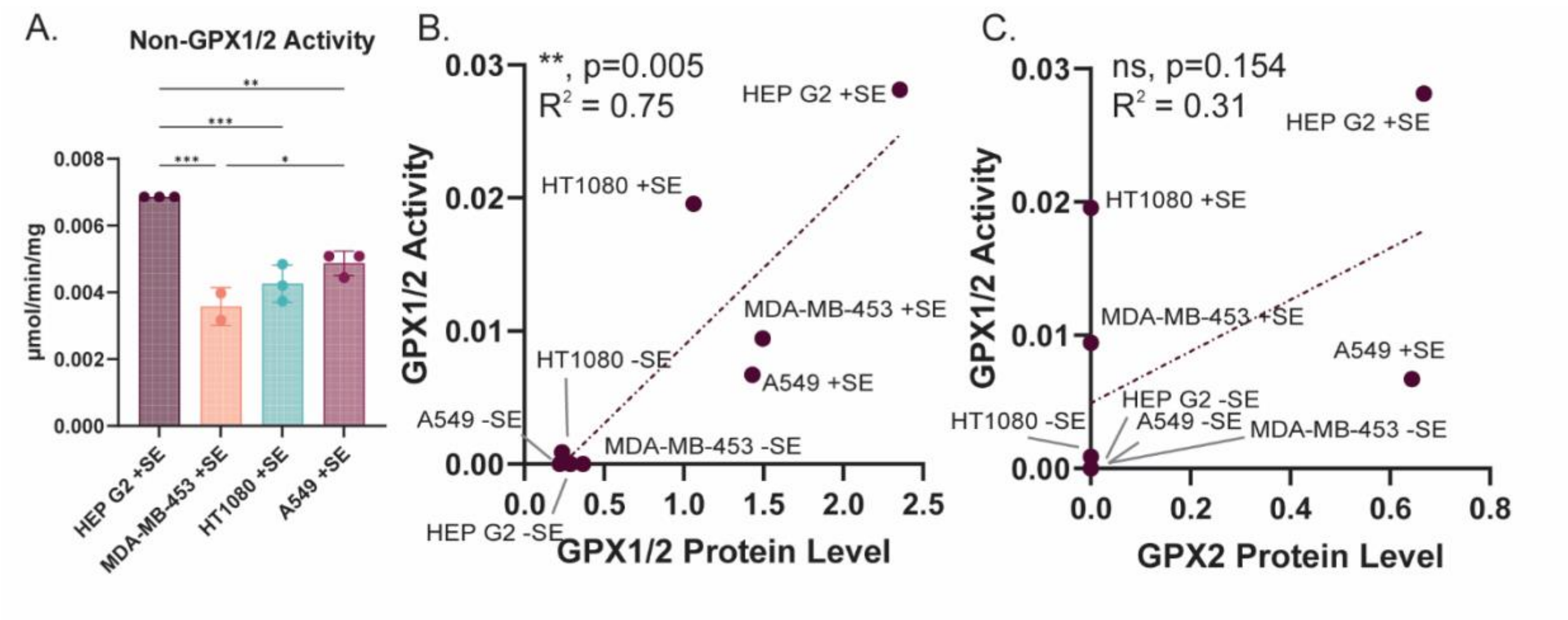

Supplementary Figure 2. (A) Non-GPX1/2 activity (uninhibited by Auranofin) from selenium-supplemented cell lines in Figure 3. Main text **Figure 3A** GPX1/2 activity correlation with **(B)** combined GPX1 and GPX2 protein levels and **(C)** GPX2 protein levels (integrated density of immunoblot bands, normalized to Actin control) determined from immunoblots in main text **Figure 3B**. P values and R^2 determined with Pearson correlation, ns= not statistically significant.

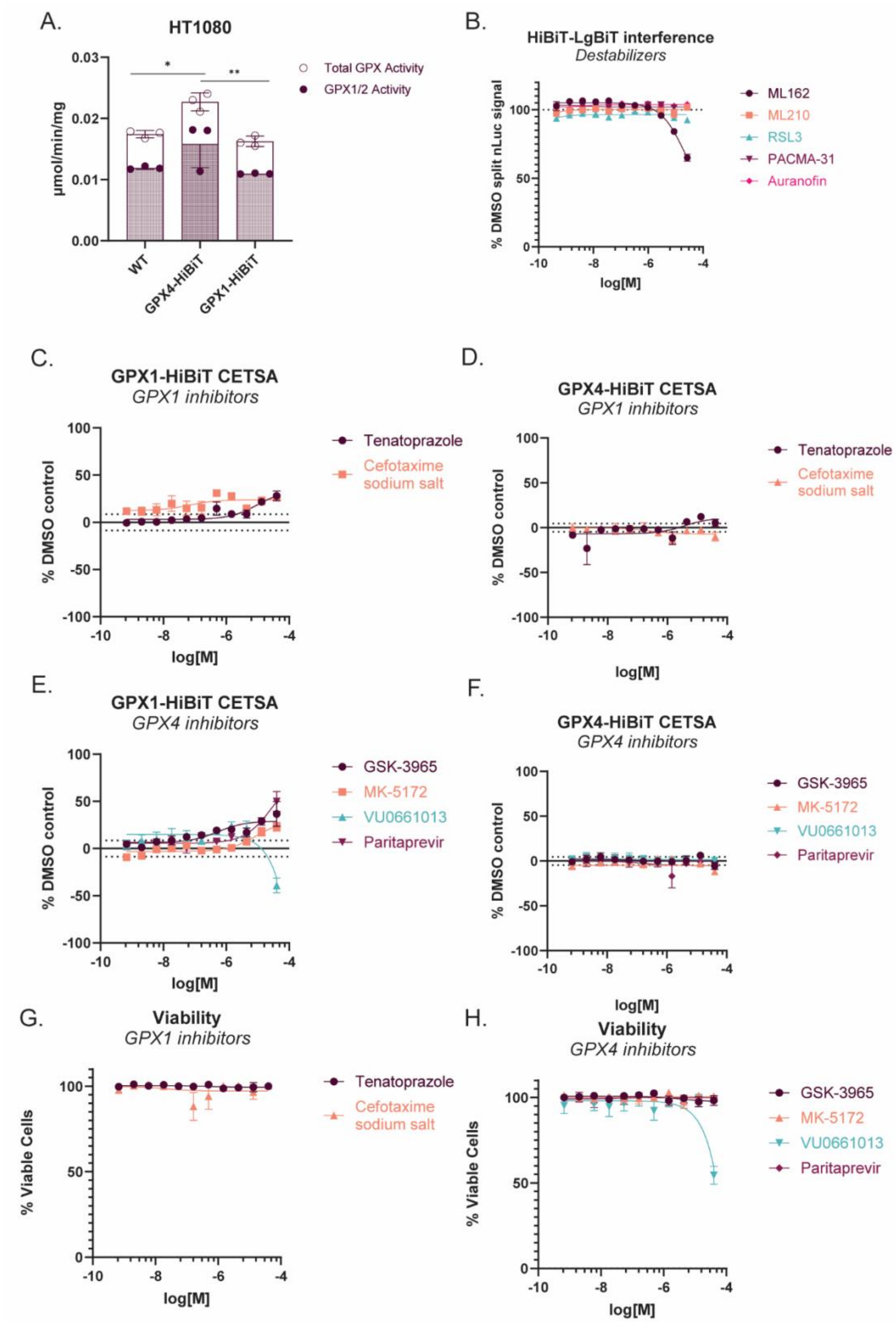

A.
HT1080
0.03
0.02
0.01
0.00
µmol/min/mg
*
**
Total GPX Activity
GPX1/2 Activity
WT
GPX4-HiBiT
GPX1-HiBiT
B.
HiBiT-LgBiT interference
Destabilizers
% DMSO split nLuc signal
log[M]
ML162
ML210
RSL3
PACMA-31
Auranofin
C.
GPX1-HiBiT CETSA
GPX1 inhibitors
% DMSO control
log[M]
Tenatoprazole
Cefotaxime sodium salt
D.
GPX4-HiBiT CETSA
GPX1 inhibitors
% DMSO control
log[M]
Tenatoprazole
Cefotaxime sodium salt
E.
GPX1-HiBiT CETSA
GPX4 inhibitors
% DMSO control
log[M]
GSK-3965
MK-5172
VU0661013
Paritaprevir
F.
GPX4-HiBiT CETSA
GPX4 inhibitors
% DMSO control
log[M]
GSK-3965
MK-5172
VU0661013
Paritaprevir
G.
Viability
GPX1 inhibitors
% Viable Cells
log[M]
Tenatoprazole
Cefotaxime sodium salt
H.
Viability
GPX4 inhibitors
% Viable Cells
log[M]
GSK-3965
MK-5172
VU0661013
Paritaprevir

Supplementary Figure 3. (A) GPX1/GPX2 (solid purple bars) superimposed on total GPX activity (white bars), the rate of NADPH consumption (μmol NADPH/min/mg), for WT, GPX1- and GPX4-HiBiT cell lysate grown in selenite-supplemented media demonstrating the HiBiT tag has no effect on GPX functionality (n=3, *$p<0.05$, **$p<0.005$). **(B)** HiBiT-LgBiT nanoLuciferase interference of compounds with a destabilizing effect in the CETSA assay. GPX1-HiBiT Ht1080 cells were dispensed onto plate pre-spotted with compound and immediately lysed for NLuc complexing (15 minutes) and read (n=2). GPX-HiBiT CETSA results for additional GPX1 biochemical inhibitors **(C and D)** and GPX4 **(D and E)** biochemical inhibitors in the GPX1-HiBiT **(C and E)** and GPX4-HiBiT **(D and F)** CETSA assay. HT1080 cells were treated for 1 hour with compound and data are normalized to DMSO controls. The standard deviation of DMSO controls is shown as a dotted line on the y-axis (n=3). **(G and H)** Viability of HT1080 cells following 1 hr. treatment with compounds from **S3C-F**. Data normalized to DMSO controls (n=4).

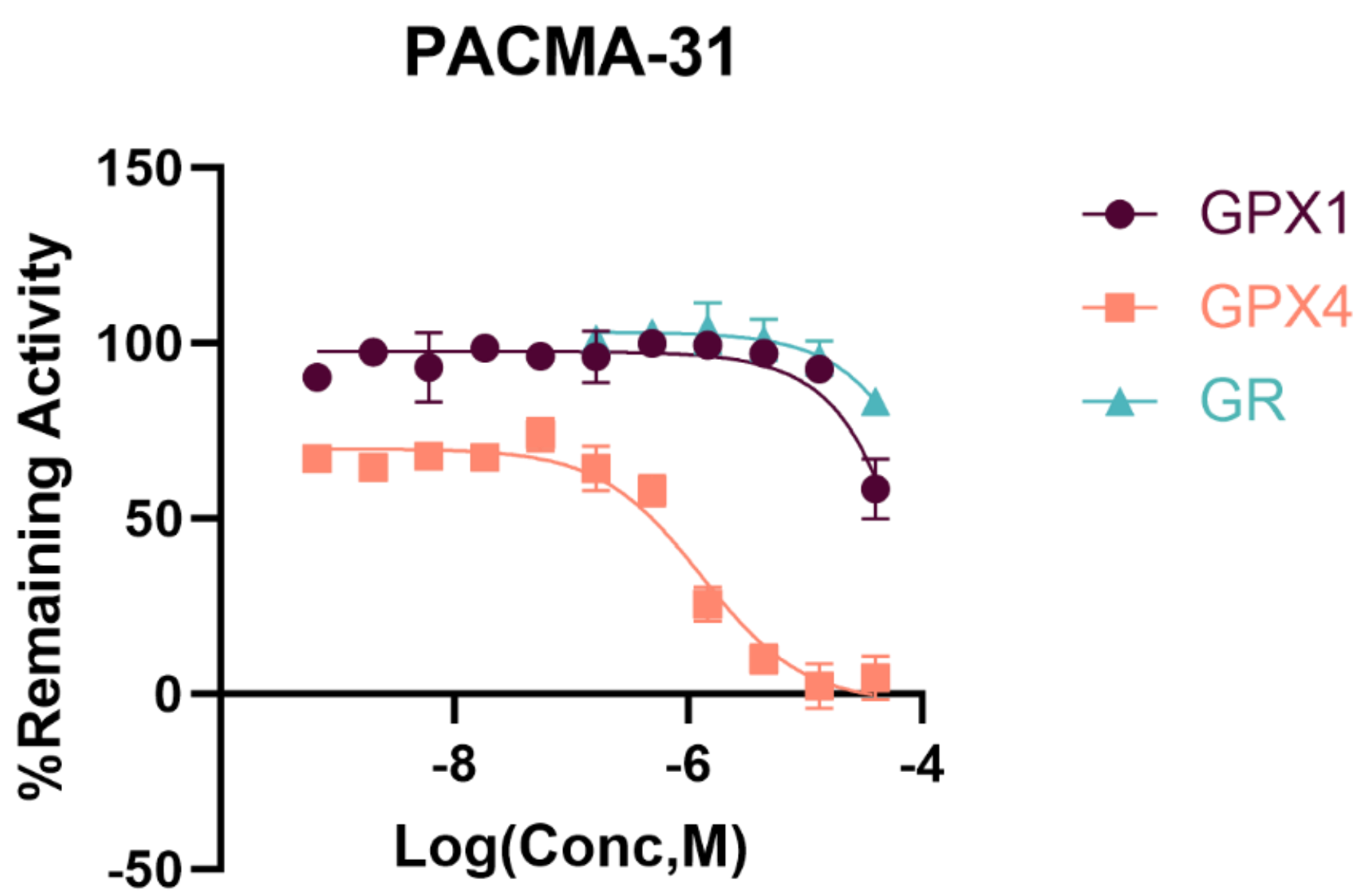

Supplementary Figure 4. PACMA-31 enzymatic inhibition of GPX1, GPX4 (IC50 = 1.3 µM) and GR (counter assay). GPX enzymatic inhibition was spectrophotometrically determined using GR-coupled consumption of NADPH and reduction of GSSG at the same final concentrations as the GR-coupled lysate activity assay. Recombinant human GPX1 (20 nM) and GPX4 (200 nM) reduced CHP with GSH co-factor, and the consumption was normalized to DMSO enzyme (100% activity) and no enzyme (0%) controls. GR counter screen (2nM GR) tested the top 6 concentrations of compound. N=2.

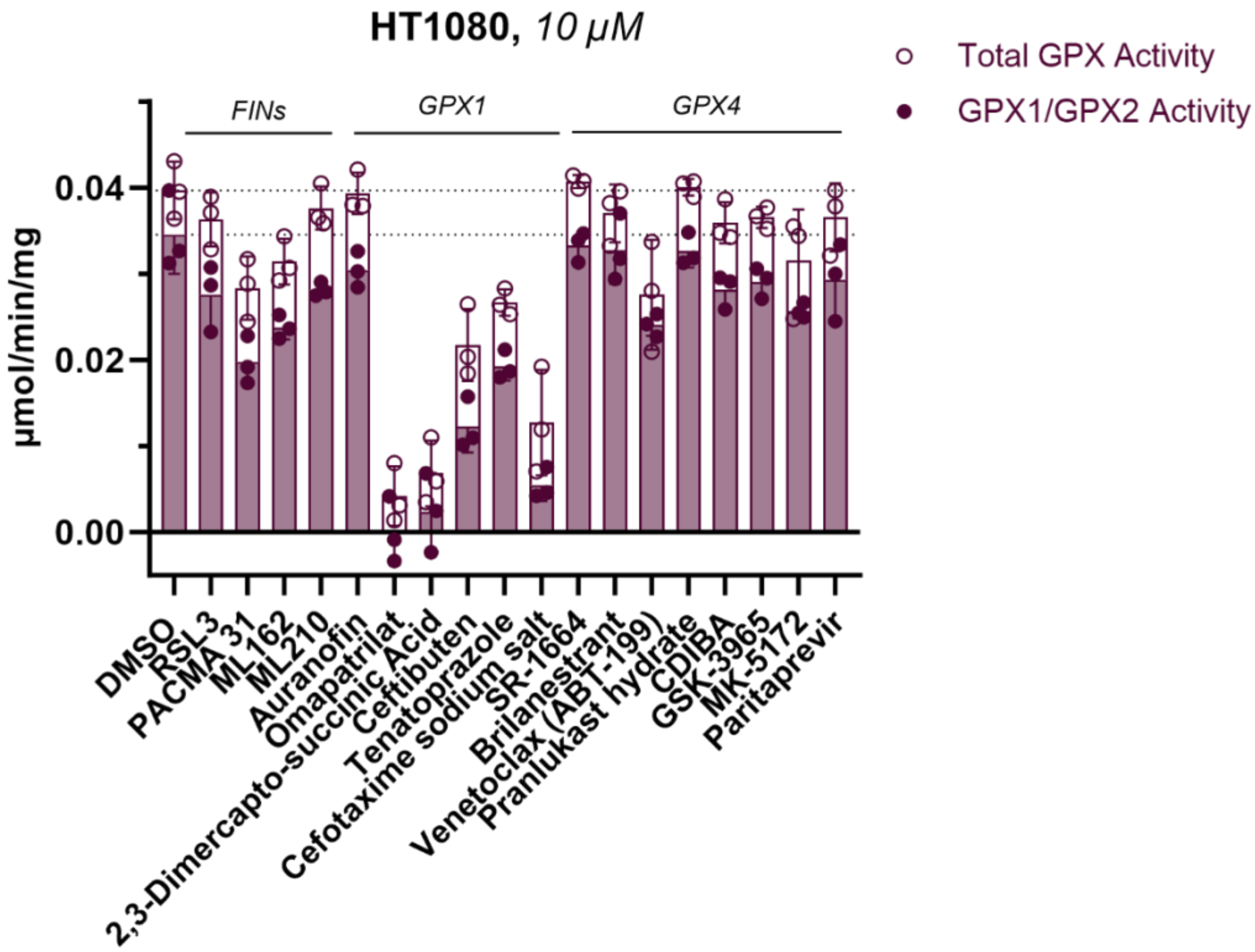

Supplementary Figure 5. HT1080 (selenium-supplemented) total GPX and GPX1/GPX2 lysate activities following 10 μM treatment with the selection of compound for this study (x-axis), which corresponds to the values reported in **Table 2** (Lysate inhibition @10 μM, **main text**). Activity is reported as μmol NADPH consumed per minute @ 340 nm absorbance, per mg lysate. Classification (text above horizontal lines) refers to direct isoenzyme inhibition profiles (**Table 2, main text**). Dashed lines represent the DMSO control total GPX (0.036 ± 0.003 μmol NADPH/min/mg) and GPX1/GPX2 (0.035 ± 0.004 μmol NADPH/min/mg) HT-1080 lysate activity. *** <0.001: MK-5172; ****<0.0001: PACMA 31, ML-162, Omapatrilat, 2,3-dimercapto-succinic acid, Ceftibuten, Tenatoprazole, Cefotaxime sodium salt, Venetoclax.
